# Rho-Kinase Inhibition Ameliorates Metabolic Disorders through Activation of AMPK Pathway in Mice

**DOI:** 10.1371/journal.pone.0110446

**Published:** 2014-11-03

**Authors:** Kazuki Noda, Sota Nakajima, Shigeo Godo, Hiroki Saito, Shohei Ikeda, Toru Shimizu, Budbazar Enkhjargal, Yoshihiro Fukumoto, Sohei Tsukita, Tetsuya Yamada, Hideki Katagiri, Hiroaki Shimokawa

**Affiliations:** 1 Department of Cardiovascular Medicine, Tohoku University Graduate School of Medicine, Sendai, Japan; 2 Department of Metabolic Diseases, Center for Metabolic Diseases, Tohoku University Graduate School of Medicine, Sendai, Japan; Institut d′Investigacions Biomėdiques August Pi i Sunyer, Spain

## Abstract

**Background:**

Metabolic disorders, caused by excessive calorie intake and low physical activity, are important cardiovascular risk factors. Rho-kinase, an effector protein of the small GTP-binding protein RhoA, is an important cardiovascular therapeutic target and its activity is increased in patients with metabolic syndrome. We aimed to examine whether Rho-kinase inhibition improves high-fat diet (HFD)-induced metabolic disorders, and if so, to elucidate the involvement of AMP-activated kinase (AMPK), a key molecule of metabolic conditions.

**Methods and Results:**

Mice were fed a high-fat diet, which induced metabolic phenotypes, such as obesity, hypercholesterolemia and glucose intolerance. These phenotypes are suppressed by treatment with selective Rho-kinase inhibitor, associated with increased whole body O_2_ consumption and AMPK activation in the skeletal muscle and liver. Moreover, Rho-kinase inhibition increased mRNA expression of the molecules linked to fatty acid oxidation, mitochondrial energy production and glucose metabolism, all of which are known as targets of AMPK in those tissues. In systemic overexpression of dominant-negative Rho-kinase mice, body weight, serum lipid levels and glucose metabolism were improved compared with littermate control mice. Furthermore, in AMPKα2-deficient mice, the beneficial effects of fasudil, a Rho-kinase inhibitor, on body weight, hypercholesterolemia, mRNA expression of the AMPK targets and increase of whole body O_2_ consumption were absent, whereas glucose metabolism was restored by fasudil to the level in wild-type mice. In cultured mouse myocytes, pharmacological and genetic inhibition of Rho-kinase increased AMPK activity through liver kinase b1 (LKB1), with up-regulation of its targets, which effects were abolished by an AMPK inhibitor, compound C.

**Conclusions:**

These results indicate that Rho-kinase inhibition ameliorates metabolic disorders through activation of the LKB1/AMPK pathway, suggesting that Rho-kinase is also a novel therapeutic target of metabolic disorders.

## Introduction

Metabolic syndrome (MetS) is a health problem caused by excessive calorie intake and low physical activity and is characterized by visceral obesity, insulin resistance and initiation of several atherogenic signs, such as hypertension, glucose intolerance and hyperlipidemia [1].

Rho-kinase is one of the effector proteins of the small GTP-binding protein RhoA, and the RhoA/Rho-kinase pathway plays an important role in various physiological cellular functions, such as vascular smooth muscle contraction, cell adhesion, motility and cytokinesis [2]. In the muscle, Rho-kinase phosphorylates the myosin-binding subunit (MBS) of myosin light-chain phosphatase (MLCPh) and inhibits its activity, resulting in muscle contraction [2]. In contrast, Rho-kinase is also one of the central mediators of inflammation, proliferation, fibrosis and apoptosis through activation of MEK/ERK, NF-κB and p38MAP kinase pathways [3], [4]. It has been previously reported that Rho-kinase is activated in metabolic syndrome in animals [5], [6] and humans [7] and that fasudil, a selective Rho-kinase inhibitor [2], exerts beneficial effects on metabolic disorders in animals [5], [6].

AMP-activated kinase (AMPK) is widely known to be a key molecule of metabolic conditions [8]. It is a hetero-trimetric protein containing α, β and γ subunits, where α subunit is the catalytic subunit [9]. The AMPK complexes containing α_2_ subunit predominate in the skeletal muscle [10], while equal levels of α_1_ and α_2_ complexes are present in the liver [11]. In the skeletal muscle, AMPK increases glucose uptake, lipid oxidation and mitochondrial biogenesis, whereas it decreases glucose production and lipid synthesis and increases lipid oxidation in the liver [9]. Indeed, AMPK has been implicated in metabolic modulation as it increases O_2_ consumption [12], glucose metabolism [13] and fatty acid oxidation [13].

Although previous reports showed that AMPK inhibits Rho-kinase activity [14], it remains to be elucidated whether Rho-kinase affects AMPK activity. In the present study, we thus aimed to examine whether Rho-kinase inhibition improves high-fat diet (HFD)-induced metabolic disorders in mice, and if so, to elucidate the involvement of AMPK pathway.

## Methods

### Ethics Statement

Animal care and the experimental procedures were approved by the Guidelines on Animal Experiments of Tohoku University and the Japanese Government Animal Protection and Management Law (No. 105–2011). All animal experiments were performed in accordance with the Guide for the Care and Use of Laboratory Animals published by the U.S. National Institute of Health (NIH Publication).

### Animal preparation

This study was approved by the Research Committee of Tohoku University Graduate School of Medicine, and the animal procedures were performed conform the NIH guidelines. C57Bl/6N mice were purchased from CREA Japan Inc. (Tokyo, Japan). AMPKα2 floxed mice, which had been backcrossed to C57Bl/6 at least 10 times, were generated as previously described [15]. DN-ROCK Tg mice, which had been backcrossed to C57Bl/6 at least 10 times, were obtained from RIKEN BioResource Center (Tsukuba, Japan) [16]. CMV-Cre mice, which had been backcrossed to C57Bl/6 at least 10 times, were obtained from Jackson Laboratory (Bar Harbor, ME, USA). AMPKα2^−/−^ mice and DN-ROCK Tg mice were generated by breeding with CMV-Cre mice and AMPKα2 floxed mice or DN-ROCK mice, respectively. All animals were housed in a room under controlled temperature (23±1°C), humidity (45–65%) and lighting with 12 hours of light and 12 hours of dark. C57Bl/6N mice were fed either a normal diet or HFD containing 60% of fat, 20% of protein and 20% of carbohydrate (D12492; Research Diet, NJ, USA) for 6 weeks. The male HFD-fed mice were simultaneously administered either vehicle or a selective Rho-kinase inhibitor, fasudil (100 mg/kg/day), a selective Rho-kinase inhibitor [3], [4], for 6 weeks. Female DN-ROCK Tg mice and littermate mice were fed HFD and vehicle. AMPKα2^−/−^ mice were fed HFD and administered either vehicle or fasudil in their drinking water for 6 weeks. As preliminary experiments, we measured the amount of drinking water of C57Bl6/N, DN-ROCK Tg and AMPKα2^−/−^ mice at 6, 9, and 12 weeks of age for 24 hours, by measuring the weight of water bottle, DS-B (Shin Factory, Fukuoka, Japan). At the end of each treatment, we measured body weight (BW) and performed glucose tolerance test (GTT) [17]. The animals were anesthetized with inhalation of isoflurane and intraperitoneal pentobarbital (50 mg/kg), humanely killed by overdose of anesthetic and cervical dislocation. We used the soleus as skeletal muscle in all experiments. Adipose weight was expressed as the total amount of epididymal and peri-renal fat.

### Metabolic Assessment

For glucose tolerance test, mice were fasted for 15 hours. Glucose (1 g/kg BW) was then injected intraperitoneally and the blood was collected from the tail vein at different time points. Oxygen consumption and carbon dioxide generation was measured by RQ-5000 as previously described [18], [19].

### Cell Culture and Drug Treatment

C2C12 skeletal muscle cells were obtained from European Collection of Cell Cultures (Salisbury, UK). Cells were grown at 37°C in 5% CO_2_ in 10% fetal bovine serum and 4.5 g/l glucose and were differentiated in DMEM containing 2% horse serum [10]. Unless otherwise stated, C2C12 myotubes were considered as myotubes after 96 hours of differentiation. C2C12 myotubes were incubated in DMEM containing 2% horse serum with hydroxyfasudil, Y27632, or dimethyl sulfoxide (DMSO) as a control. NAD^+^/NADH ratio was measured after 48 hours incubation in DMEM containing 2% horse serum with hydroxyfasudil and/or compound C or DMSO by NAD^+^/NADH quantitative kit (Biovision Research Products, CA, USA).

### Western Blot Analysis

Western blot analysis was performed using antibodies that specifically recognize proteins, including AMPKα (Cell Signaling Technology, MA, USA), phospho-AMPKα at Thr172 (p-AMPK, Cell Signaling Technology, MA, USA), acetyl CoA carboxylase (ACC, Cell Signaling Technology), phospho-ACC at Ser79 (Cell Signaling Technology), myosin phosphatase targeting subunit-1 (MYPT-1) (BD Biosciences, CA, USA), phospho-MYPT-1 at Thr696 (p-MYPT1, Cell Signaling Technology), ROCK1 (BD Biosciences, CA, USA) and ROCK2 (BD Biosciences). The same amount of extracted protein (10∼20 µg) was loaded for sodium dodecyl sulphate-polyacrylamide gel electrophoresis (SDS-PAGE) immunoblot analysis. After incubating with HRP-conjugated anti-mouse (Sigma Aldrich, St. Louis, MO, USA) or anti-rabbit IgG antibody (Cell Signaling, Danvers, MA, USA), the regions containing proteins were visualized using ECL prime Western blotting detection system (Amasham Biosciences, Buckinghamshire, UK). Each band was normalized by corresponding value of α-tubulin as an internal control. Densitometric analysis was performed by Image J (NIH) Software [20].

### Mitochondrial DNA Copy Number

For the quantification of mitochondrial DNA copy number, real-time PCR analysis was performed with the NovaQUANT Mouse Mitochondrial to Nuclear DNA Ratio Kit (Novagen, Darmstadt, Germany) according to the manufacturer's instructions[21]. DNA extractions were performed on frozen mouse striatum using a QIAamp DNA mini kit (Qiagen, CA, USA).

### RNA Extraction and Semi-quantitative Real-Time PCR

Total RNA was extracted from the liver and skeletal muscle with the RNeasy universal mini kit (Qiagen, CA, USA). The samples were crushed and total RNA was extracted with the RNeasy universal mini kit (Qiagen,CA, USA). We composed complementary DNA using a reverse transcriptase from RNA promptly. Complementary DNA was stored at −80°C and used within a week. From C2C12 myotubes, total RNA was extracted with the RNeasy plus mini kit (Qiagen, CA, USA). Total RNA was subjected to reverse transcription by using PrimeScript (Takara Bio Inc., Shiga, Japan) according to the manufacturer's protocol. Semi-quantitative real-time PCR was performed with a CFX96 Real-Time PCR Detection System (Bio-Rad Lab., CA, USA) using SYBR Premix EX Taq (Takara Bio Inc., Shiga, Japan). mRNA expression was normalized to that of *Gapdh*.

### siRNA Transfection in C2C12 Myotubes

Multiple siRNA duplexes for ROCK1 and ROCK2 were purchased from Qiagen (CA, USA) (Table S1). C2C12 myotubes were transfected with the reagent (HiPerFect Transfection Reagent; Qiagen, CA, USA) with either 20 nmol/L control siRNA or 20 nmol/L siRNA specific for ROCK1, ROCK2, LKB1 and TAK1. A functional non-targeting siRNA (Qiagen, CA, USA) was used as a control. After 96 hours of post-transfection, the cells were analyzed by Western blot or real-time polymerase chain reaction (PCR).

### AMPK Kinase Assay

C2C12 myotubes were treated with or without fasudil for 6 hours and cells were lysed in lysis buffer (20 mM Tris-HCl, pH7.5, 150 mM NaCl, 1% Triton, 10% glycerol, 1 mmol/L EDTA, 1 mmol/L EGTA and protease inhibitor cocktail). Immunoprecipitation of the AMPKα1 and AMPKα2 protein were performed using µMACS™ and Multi-MACS™ Protein G Kits (Miltenyi Biotec GmbH, Bergisch Gladbach, Germany). Lysate were resuspended in 100 ml AMPK kinase assay buffer (Cyclex, CY-1182). After 30 min incubation at 30°C with a substrate peptide, IRS-1 S789, AMPK activity was analyzed by measuring the phosphorylation level of IRS-1 S789, according to the manufacturer's instructions.

### Statistical Analysis

Comparisons of parameters between two groups were performed with unpaired Student's t-test. Statistical analysis was analyzed by one-way ANOVA followed by Bonferroni/Dunn's post-hoc test for multiple comparisons. Statistical significance was evaluated with IBM SPSS statistics ver. 21 (IBM, NY, USA). A P value of<0.05 was considered to be statistically significant.

## Results

### Pharmacological Inhibition of Rho-kinase Improves Metabolic Phenotypes in High-fat Diet-fed Mice

**Figure 1:**
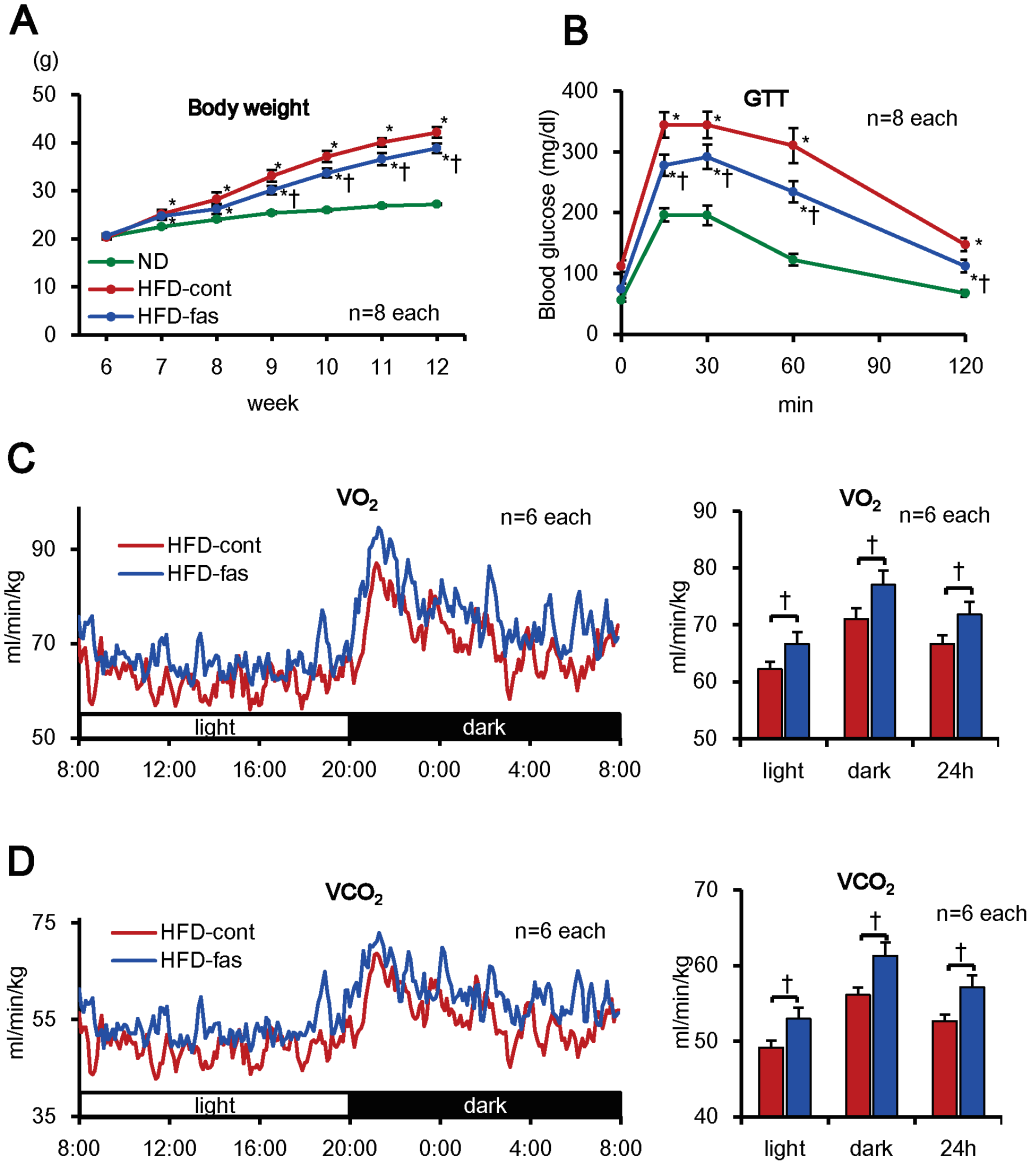
Fasudil Ameliorates Metabolic Phenotypes in HFD-fed Mice. (**A**) Body weight was measured in the normal diet (ND), HFD-cont and HFD-fas groups from 6 to 12 weeks of age. It was significantly increased in the HFD-cont group since 7 weeks of age, compared with normal diet group and was significantly suppressed in the HFD-fas group since 9 weeks of age compared with the HFD-cont group (male mice) (n = 8 each). (**B**) Glucose tolerance test at 12 weeks of age showed that the response was improved in the HFD-fas group compared with the HFD-cont group (n = 8 each). (**C, D**) O_2_ consumption and CO_2_ generation were measured at 12 weeks of age. Both O_2_ consumption and CO_2_ generation were significantly increased in the HFD-fas group throughout the day compared with the HFD-cont group (n = 6 each). Results are expressed as mean ± SEM. *P<0.05 vs. normal diet (ND) group. ^†^P<0.05 vs. HFD-cont group. ^§^P<0.05.

We first examined whether fasudil suppresses HFD-induced metabolic disorders in wild-type mice. The HFD group was simultaneously received either vehicle (HFD-cont group) or fasudil (100 mg/kg/day, HFD-fas group). In the skeletal muscle, liver and white adipose tissue, the extent of MYPT phosphorylation, a marker of Rho-kinase activity, was increased in the HFD-cont group compared with the ND group and was inhibited in the HFD-fas group (**Fig. S1A**). Body weight was significantly increased in the 2 HFD groups since 7 weeks of age, which was significantly suppressed in the HFD-fas group since 9 weeks of age (**Fig. 1A**), despite the comparable food intake among the 3 groups (**Fig. S1B**). The weight of white adipose tissue (WAT) and the diameter of adipocytes in brown adipose tissue (BAT) were also increased in the 2 HFD groups and were significantly decreased in the HFD-fas group (**Table 1**, **Fig. S2A,B**). Similarly, glucose tolerance was impaired in the HFD-cont group, which was improved in the HFD-fas group (Fig. 1B, Fig. S1C). Serum lipid levels (total cholesterol, LDL-C, HDL-C) and serum leptin level were significantly increased in the 2 HFD groups, which was suppressed in the HFD-fas group (**Table 1**). Furthermore, both O_2_ consumption and CO_2_ generation were significantly increased in the HFD-fas group compared with the HFD-cont group at 8 weeks of age (**Fig. 1C,D**), whereas body weight, locomotor activity and respiration quotient (RQ) were unaltered (**Fig. S3A-C**). These results suggest that Rho-kinase is involved, at least in part, in HFD-induced metabolic disorders, including weight gain, glucose intolerance and reduced energy consumption.

**Table 1:** Metabolic Parameters of Wild-type Mouse Groups.

	ND	HFD-cont	HFD-fas
White adipose tissue weight (mg)	428.0±40.8	3301.5±70.0^*^	2879.9±132.5^*†^
WAT weight/body weight (mg**/**g)	17.2±1.55	84.5±1.60^*^	78.2±2.94^*†^
Total cholesterol (mg/dl)	75.9±3.8	145.4±7.7^*^	118.9±8.7^*†^
LDL-C (mg/dl)	9.9±0.8	41.9±5.5^*^	26.4±4.7^*†^
HDL-C (mg/dl)	64.5±3.2	102.1±2.7^*^	90.8±4.6^*†^
Triglyceride (mg/dl)	14.8±1.5	12.1±1.1	8.5±0.9^*†^
HOMA-IR	0.10±0.02	0.89±0.10^*^	0.53±0.14^*†^
Leptin (ng/ml)	1.7±0.4	40.4±1.9^*^	34.9±2.4^*†^

Results are expressed as mean ± SEM. The data were obtained at 12 weeks of age after 6-week treatment, with 15 h of fasting (n = 8 each). HOMA-IR was calculated using the following formula: {*fasting glucose (mg/dl) × fasting insulin (ng/ml)/405*}. Adipose tissue weight was the sum of the epididymal and peri-renal fat. ND, normal diet; HFD, high-fat diet. *P<0.05 vs. ND group, ^†^P<0.05 vs. HFD-cont group.

**Figure 2:**
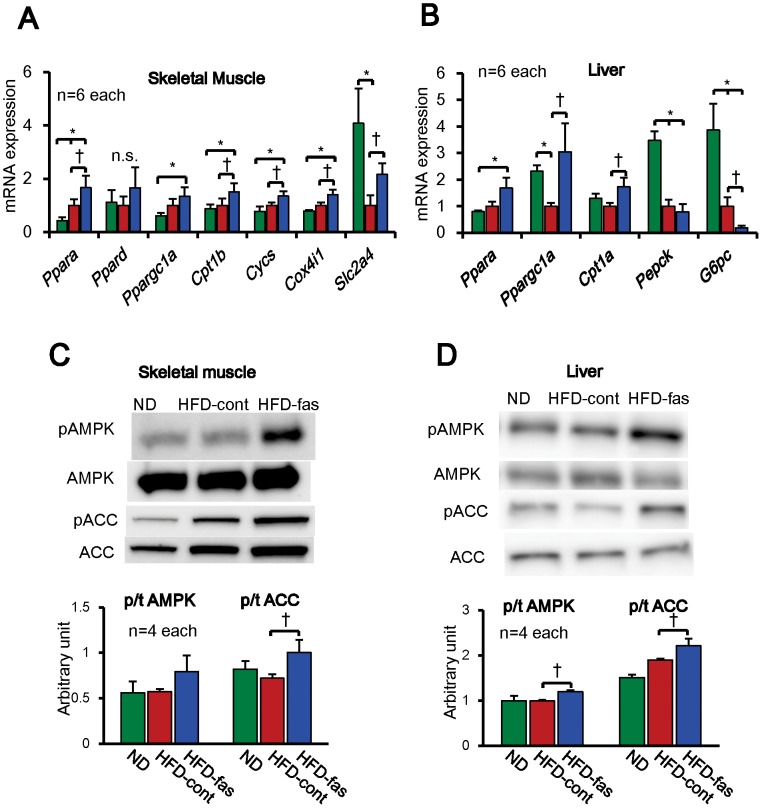
Fasudil Increases mRNA Expression of Molecules Related to Energy Metabolism and Activates AMPK. (**A, B**) mRNA expression of the molecules that are linked to fatty acid oxidation (*Ppara*, *Ppard* and *Cpt1b*), mitochondrial energy production (*Cycs* and *Cox4i1*) and glucose transporter (*Slc2a4*) were measured by real-time RT-PCR in the skeletal muscle and liver (n = 6 each). (**C, D**) Representative Western blots of the skeletal muscle and liver for total and phosphorylated forms of AMPK and ACC (n = 4 each). The extent of AMPK and ACC phosphorylation, as markers of AMPK activation, were significantly enhanced by the fasudil treatment (n = 4 each). Results are expressed as mean ± SEM. *P<0.05.

To elucidate the mechanisms of the increased energy metabolism by Rho-kinase inhibition, we then examined mRNA expressions of the molecules that are related to energy metabolism, including fatty acid oxidation, mitochondrial function and glucose metabolism. In the skeletal muscle, mRNA expression of the molecules that are related to fatty acid oxidation (*Ppara* and *Cpt1b*), mitochondrial energy production (*Cycs* and *Cox4i1*) and glucose transporter (*Slc2a4*) were all significantly increased in the HFD-fas compared with the HFD-cont group, whereas *Ppard* was unaltered among the 3 groups (**Fig. 2A**). Similarly, in the liver, mRNA expressions of the molecules that are linked to fatty acid oxidation (*Cpt1b*) and mitochondrial biogenesis (*Ppargc1a*) were increased and that of gluconeogenesis (*G6pc*) was decreased in the HFD-fas compared with the HFD-cont group (**Fig. 2B**). Furthermore, mRNA expression of *Ppargc1a* and *Ucp1* were increased in the HFD-fas group compared with the HFD-cont group in BAT (**Fig. S2C**). Since AMPK activation is known to be involved in *Cpt1b*, *Cycs*, *Cox4i1* and *Slc2a4* expression, we next examined AMPK activity. As a marker of AMPK activity, we examined the extent of AMPK phosphorylation at Thr172 and that of acetyl CoA carboxylase (ACC) phosphorylation at Ser79. Although in the skeletal muscle, the extent of AMPK phosphorylation only tended to be increased, that of ACC phosphorylation was significantly increased in the HFD-fas compared with the HFD-cont group (**Fig. 2C**). In contrast, in the liver, the extents of both AMPK and ACC phosphorylations were significantly increased in the HFD-fas compared with the HFD-cont group (**Fig. 2D**).

### Genetic Inhibition of Rho-kinase Ameliorates Metabolic Phenotypes in High-fat Diet-fed Mice

**Figure 3:**
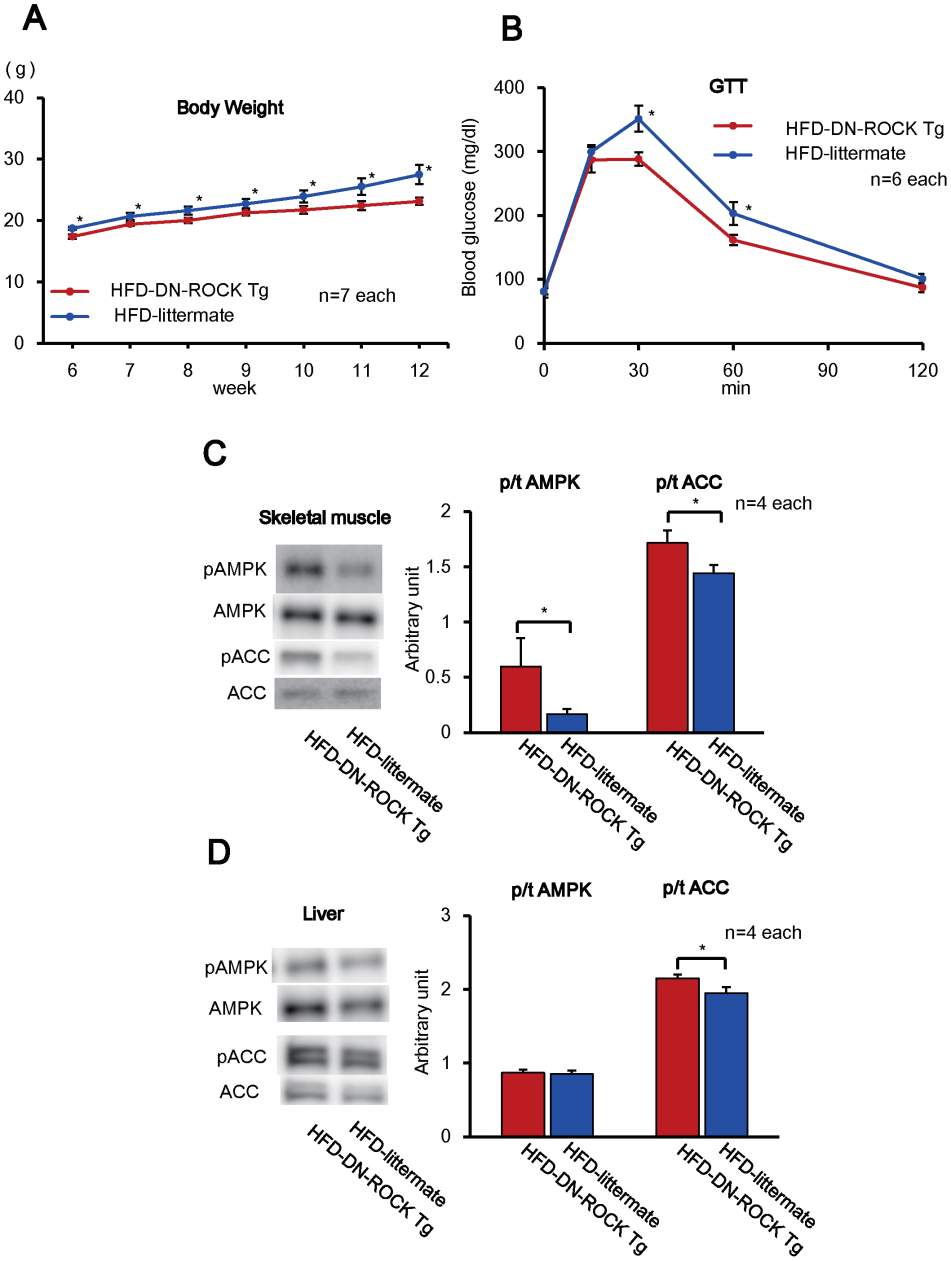
Improved Metabolic Phenotypes in Mice with Systemic Overexpression of Dominant-Negative Rho-kinase. (**A**) The increase in body weight was significantly suppressed in the HFD-DN-ROCK Tg group compared with the HFD-littermate group (female) (n = 7 each). (**B**) Glucose tolerance test at 12-weeks of age showed that the responses were improved in the HFD-DN-ROCK Tg group compared with the HFD-littermate group (n = 6 each). (**C, D**) In the skeletal muscle (**C**) and the liver (**D**), the extent of AMPK phosphorylation and that of ACC phosphorylation were significantly increased in the HFD-DN-ROCK Tg mice compared with the littermate mice (n = 4 each). Results are expressed as mean ± SEM. *P<0.05.

Next, we assessed the metabolic phenotypes in HFD-fed mice, using systemic overexpression of dominant-negative Rho-kinase (DN-ROCK) mice and their littermate mice (littermate) (**Fig. S4A**). Compared with the littermate, Rho-kinase activity, as evaluated by the extent of MYPT-1 phosphorylation, was reduced approximately 30% in the liver, skeletal muscle and white adipose tissue in DN-ROCK mice (**Fig. S4B**). In HFD fed DN-ROCK mice, the increase in body weight was significantly suppressed compared with HFD-littermates from 6 to 12 weeks of age (**Fig. 3** **A**) and glucose tolerance was improved (**Fig. 3B**, **Fig. S4D**), despite the comparable food intake in the 2 group (**Fig. S4C**). The weight of white adipose tissue (WAT) and the diameter of adipocytes in brown adipose tissue (BAT) were significantly decreased in DN-ROCK mice (**Table 2**, **Fig. S5A,B**). Similar to wild-type mice, mRNA expression of *Ppargc1a* and *Ucp1* in BAT were increased in HFD-fed DN-ROCK mice compared with HFD-fed littermate mice (**Fig. S5C**). Serum lipid levels (total cholesterol, LDL-C, HDL-C) and serum leptin level were significantly increased in the HFD-littermate group (**Table 2**). In the skeletal muscle, the extent of AMPK phosphorylation and that of ACC phosphorylation were significantly increased in the DN-ROCK compared with the littermates (**Fig. 3C**). In contrast, in the liver, the extent of ACC phosphorylation was significantly increased in the DN-ROCK mice compared with the littermates, whereas that of AMPK phosphorylation only tended to be increased (**Fig. 3D**). In addition, the ratio of mitochondrial DNA and nuclear DNA, a marker of mitochondrial number, was increased in the skeletal muscle of HFD-fed DN-ROCK mice (**Fig. S5D**). These results indicate that genetic inhibition of Rho-kinase also suppresses HFD-induced metabolic phenotypes, via AMPK activation.

**Table 2:** Metabolic parameters of HFD-DN-ROCK Tg group and HFD-littermate group.

	HFD-DN-ROCK Tg	HFD-littermate
White adipose tissue weight (mg)	632.7±231.0	1619.3±562.1^*^
WAT weight/Body Weight (mg/g)	25.1±7.45	52.6±13.6^*^
Total cholesterol (mg/dl)	63.2±5.5	80.7±3.5^*^
LDL-C (mg/dl)	11.4±1.0	15.5±1.3^*^
HDL-C (mg/dl)	49.6±4.4	63.5±2.5^*^
Triglyceride (mg/dl)	10.3±1.8	11.7±0.5
HOMA-IR	0.06±0.02	0.18±0.05^*^
Leptin (ng/ml)	4.0±2.0	33.8±20.3^*^

Results are expressed as mean ± SEM. The data were obtained at 12-week-old, with 15 h of fasting (n = 6 each). HOMA-IR was calculated using the following formula: {*fasting glucose (mg/dl) × fasting insulin (ng/ml)/405*}. Adipose tissue weight was the sum of the epididymal and peri-renal fat. *P<0.05 vs. HFD-DN-ROCK Tg group.

### Lack of the Effects of Pharmacological Inhibition of Rho-kinase on Body Weight and Energy Consumption in AMPKα2-deficient Mice

**Figure 4:**
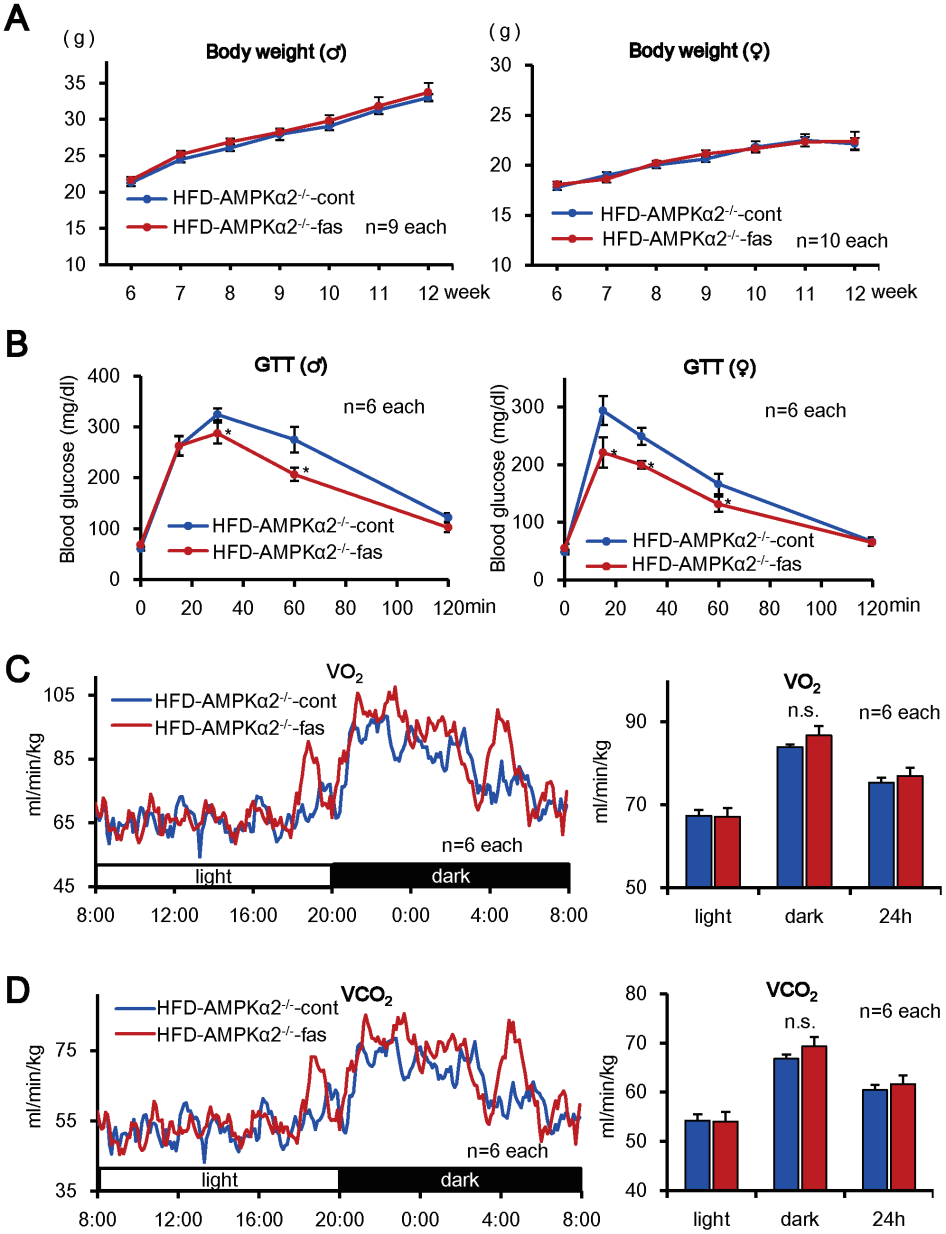
No Effects of Fasudil on Metabolic Phenotypes in AMPKα2^−/−^ Mice Except for Glucose Metabolism. (**A**) Body weight was similarly increased in both the HFD-AMPKα2^−/−^-cont and HFD-AMPKα2^−/−^-fas groups in both male (left) and female (right) (n = 9 each). (**B**) Glucose tolerance performed at 12 weeks of age was improved in the HFD-AMPKα2^−/−^-fas group compared with the HFD-AMPKα2^−/−^-cont group in both male (left) and female (right) (n = 10 each). (**C, D**) VO_2_ and VCO_2_ were comparable throughout the day in female mice (n = 6 each). Results are expressed as mean ± SEM. *P<0.05 vs. HFD-AMPKα2^−/−^- cont group.

**Figure 5:**
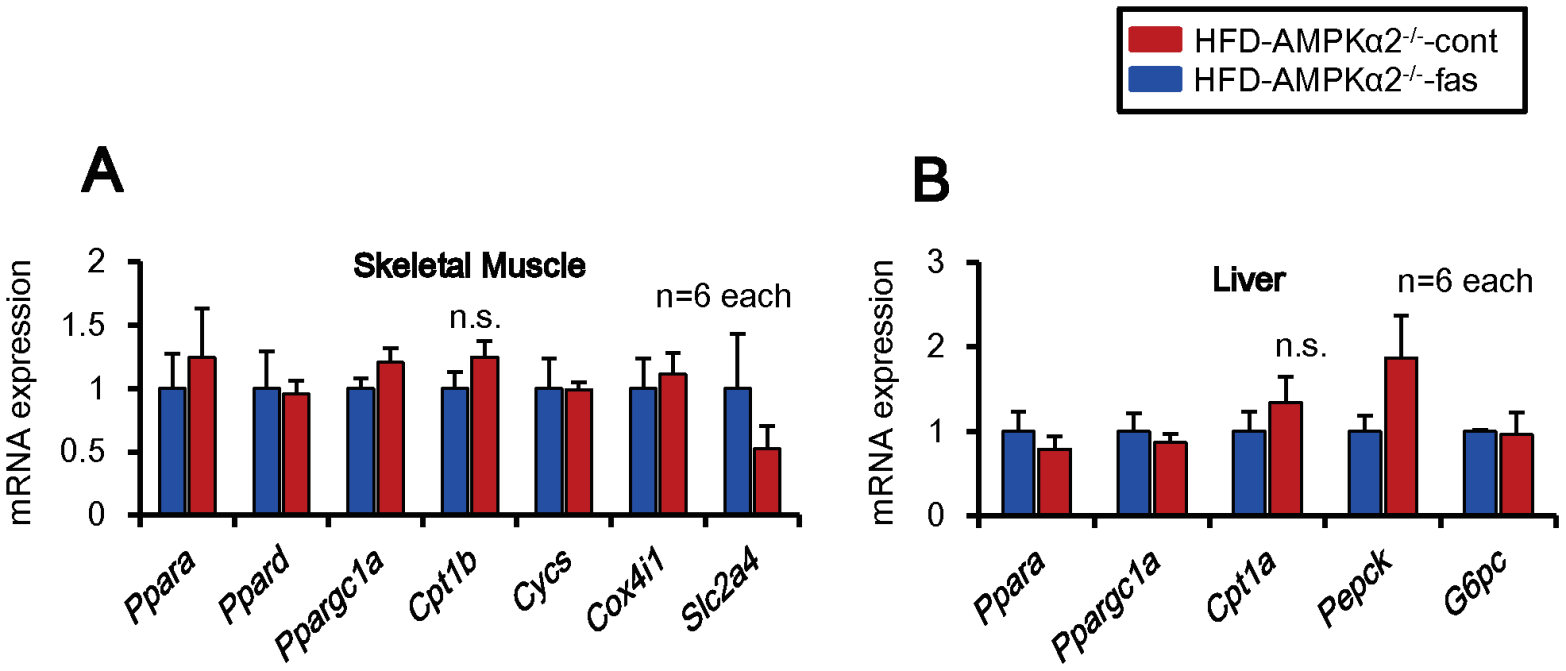
No Effects of Fasudil on mRNA Expression of Molecules Related to Energy Metabolism in AMPKα2^−/−^ Mice. mRNA expression of the molecules related to fatty acid oxidation (*Ppara*, *Ppard* and *Cpt1b*), mitochondrial energy production (*Cycs* and *Cox4i1*) and glucose transporter (*Slc2a4*) were examined by real-time RT-PCR in the skeletal muscle and liver. In the skeletal muscle (**A**) and liver (**B**), the mRNA expressions were unaltered in both groups (male mice) (n = 6 each). Results are expressed as mean ± SEM.

Since we found that Rho-kinase inhibition increases AMPK activity, we next examined whether AMPKα2 is involved in the interaction between Rho-kinase and AMPK using AMPKα2-deficient (AMPKα2^−/−^) mice (**Fig. S6A**). In HFD-fed AMPKα2^−/−^ mice, although fasudil had no inhibitory effects on the increases in body weight (**Fig. 4** **A**), WAT weight (**Table 2**), the diameter of adipocytes in BAT (**Fig. S8A,B**), food intake (**Fig. S6B**) or serum lipid profiles (**Table 3**), it improved glucose tolerance (**Fig. 4B**, **Fig. S6C**). Furthermore, fasudil had no effects on O_2_ consumption (**Fig. 4C**), CO_2_ production (**Fig. 4D**), body weight (**Fig. S7A**), locomotor activity (**Fig. S7B**) or respiration quotient (**Fig. S7C**) in AMPKα2^−/−^ mice. Fasudil had no effect on mRNA expression of the molecules related to energy metabolism in the skeletal muscle and liver of HFD-fed AMPKα2^−/−^ mice (**Fig. 5A,B**). In addition, the ratio of mitochondrial DNA and nuclear DNA, a marker of mitochondrial number, was unaltered in the skeletal muscle with or without fasudil (**Fig. S8D**). Similarly, fasudil had no effect on mRNA expression of the molecules related to energy metabolism in the skeletal muscle, liver or BAT of HFD-fed AMPKα2^−/−^ mice (**Fig. 4A,B**, **Fig. S8C**).

**Table 3:** Metabolic Parameters of HFD-AMPKα2^−/−^ Mouse Groups.

	HFD-AMPKα2^−/−^-cont	HFD-AMPKα2^−/−^-fas
White adipose tissue weight (mg)	1945.4±250.5	2165.7±283.5
WAT weight/body weight (mg/g)	61.2±6.04	66.5±7.83
Total cholesterol (mg/dl)	101.9±8.6	108.2±7.7
LDL-C (mg/dl)	14.7±1.7	14.3±2.3
HDL-C (mg/dl)	84.0±6.8	91.0±6.0
Triglyceride (mg/dl)	16.7±4.4	13.7±1.9
HOMA-IR	0.33±0.08	0.18±0.03^*^
Leptin (ng/ml)	33.0±16.7	32.9±15.5

Results are expressed as mean ± SEM. The data were obtained at 12 weeks of age after 6-week treatment, with 15 h of fasting (n = 6 each). HOMA-IR was calculated using the following formula: {*fasting glucose (mg/dl) × fasting insulin (ng/ml)/405*}. Adipose tissue weight was the sum of the epididymal and the peri-renal fat. *P<0.05 vs. HFD-AMPKα2^−/−^- cont group.

### Rho-kinase Inhibition Activates AMPK in Skeletal Muscle Cells

**Figure 6:**
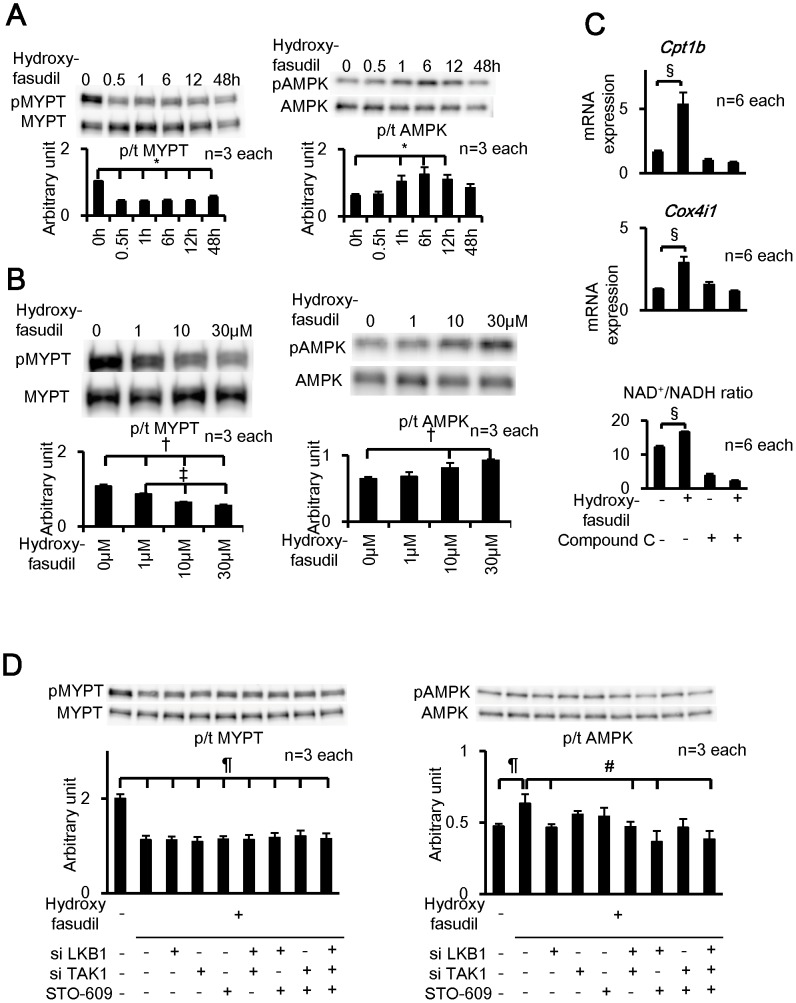
Fasudil Activates AMPK in Vitro via LKB1 Pathway. (**A**) In C2C12 myotubes, hydroxyfasudil (30 µmol/L) increased AMPK phosphorylation with a peak at 6 h after incubation (n = 3 each). (**B**) In C2C12 myotubes, hydroxyfasudil increased AMPK phosphorylation in a concentration-dependent manner after 6 hours incubation (n = 3 each). (**C**) Hydroxyfasudil (10 µmol/L for 48 h) significantly up-regulated mRNA expression of *Cpt1b* and *Cox4il* as well as NAD^+^/NADH ratio, all of which were inhibited by compound C (50 µmol/L) (n = 6 each). Results are normalized by the expression of *Gapdh*. (**D**) AMPK activation by hydroxyfasudil was significantly inhibited by siRNA for LKB1 and there was no additional effect by TAK1 or STO-609 inhibition (n = 3 each). Results are expressed as mean ± SEM. *P<0.05 vs. 0 h, †P<0.05 vs. 0 µM, ‡P<0.05 vs. 1 µM, §P<0.05 vs. fasudil without compound C, ¶P<0.05 vs. without hydroxyfasudil, siLKB1, siTAK1 and STO-609, #P<0.05 vs. hydroxyfasudil.

We then examined the mechanisms of AMPK activation by Rho-kinase inhibition in cultured skeletal muscle cell line (C2C12 cells). We used hydroxylfasudil, an active metabolite of fasudil, and another Rho-kinase inhibitor, Y27632, both of which inhibit the 2 Rho-kinase isoforms, ROCK1 and ROCK2, in a competitive manner [22]. Rho-kinase inhibition by hydroxyfasudil (30 µmol/L) was noted as early as 30 min after administration and lasted for 48 hours, whereas its activating effect on AMPK peaked at 6 hours after administration (**Fig. 6A**). The concentration-dependent effects of hydroxyfasudil on Rho-kinase and AMPK activities were also noted (**Fig. 6B**). The similar time-dependent and dose-dependent effects were also noted with Y27632 (10 µmol/L) (**Fig. S9A,B**). We next examined which isoform of AMPKα (α1 or α2) was involved in the Rho-kinase pathway. Using immnoprecipitation with AMPKα1 and AMPKα2 antibody in C2C12 myotubes, we measured AMPK activity and found that AMPK activities were significantly increased by the hydroxyfasudil treatment in samples from whole cell lysate and immnoprecipitated with AMPKα2 antibody, but not in those immnoprecipitated with AMPKα1 (**Fig. S10A,B**).

We examined whether AMPK activation by hydroxyfasudil activate the downstream targets of AMPK. mRNA expression of *Cpt1b* and *Cox4i1*, both of which are known to be activated by AMPK, were significantly increased in C2C12 cells after 48 hours of incubation with hydroxyfasudil and was completely suppressed by an AMPK inhibitor, compound C (**Fig. 6C**). Furthermore, the NAD^+^/NADH ratio, a functional marker of AMPK activation, was significantly increased by hydroxyfasudil, which was again significantly inhibited by compound C (**Fig. 6C**). To confirm the association between Rho-kinase and AMPK in vitro, we further examined the inhibitory effects of siRNAs for ROCK1 and ROCK2 (**Fig. S11A**). When combined, the siRNAs for ROCK1 and ROCK2 significantly inhibited Rho-kinase activity and increased AMPK activity (**Fig. S11B**), similar to hydroxyfasudil. Knockdown of both ROCK1 and ROCK2 by siRNA also significantly increased mRNA expression of *Cpt1b* and *Cox4i*, which was also abolished by compound C (**Fig. S11C**).

### Rho-kinase Inhibition Activates AMPK Through LKB1, an Upstream Kinase of AMPK

**Figure 7:**
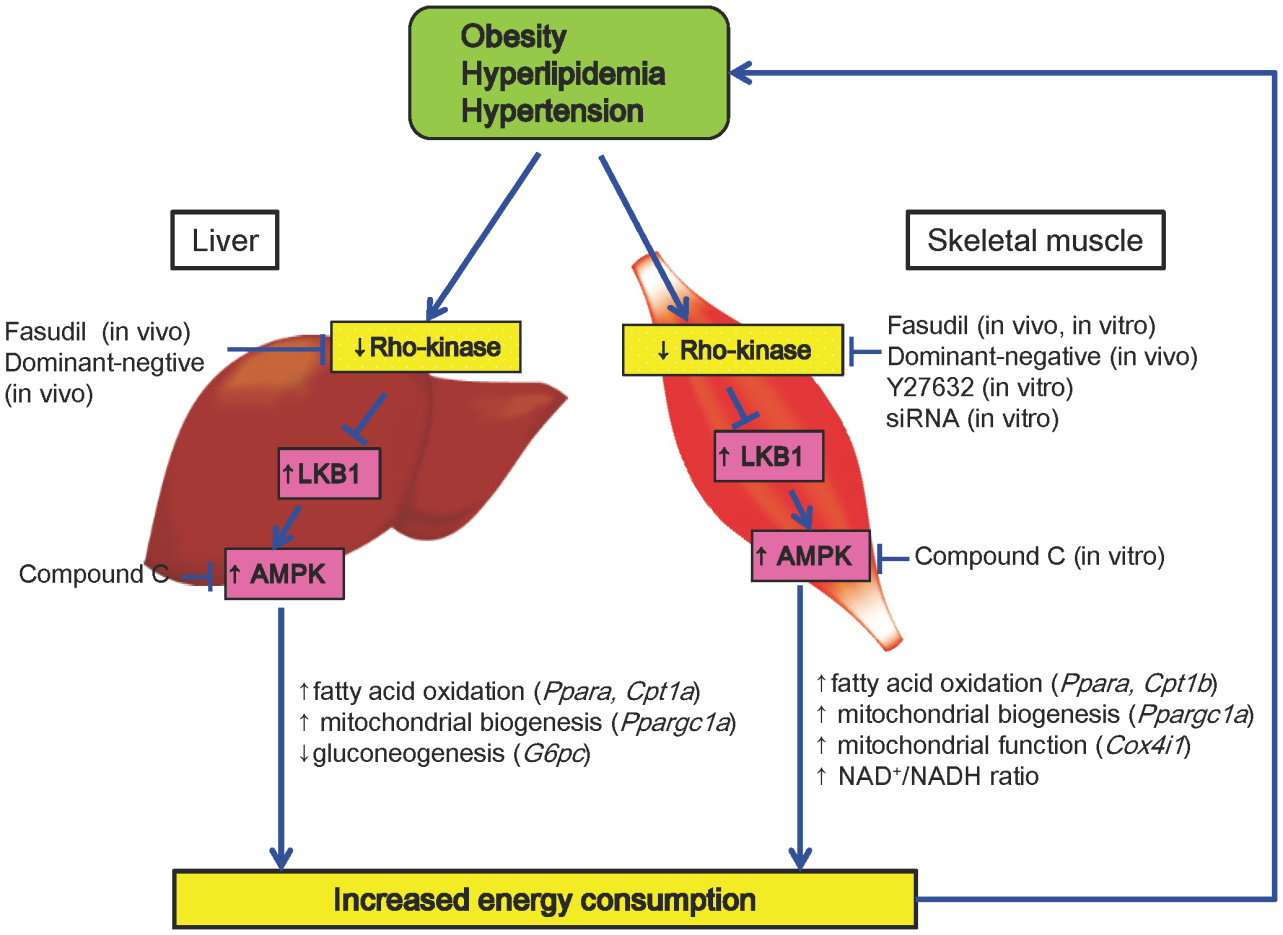
Summary of the Present Study. Rho-kinase inhibition activates AMPKα2 via LKB1 pathway with a resultant increase in energy consumption and improvement of metabolic disorders (e.g. hypertension, obesity and hyperlipidemia). Although Rho-kinase inhibition also improves insulin tolerance, this might not be mediated by AMPK activation, at least in the present study.

Finally, we examined the molecular mechanisms of AMPK activation by Rho-kinase inhibition in C2C12 cell. AMPK is known to have 3 up-stream kinases, including liver kinase 1 (LKB1) [23], Ca^2+^/calmodulin-dependent protein kinase kinase β (CaMKKβ) [24] and transforming growth factor-β-activating kinase 1 (TAK1) [25]. STO-609 (a CaMKKβ inhibitor), oxozeaenol (a TAK1 inhibitor) or siRNA for TAK1 (**Fig. S12B**) had no effects on the AMPK activation by hydroxylfasudil (**Fig. S13B**, **Fig. S14A,B**). In contrast, siRNA for LKB1 completely suppressed the AMPK activation by hydroxyfasudil (**Fig. S12A**, **Fig. S13A**). We further examined the combined effects of siRNA for LKB1 and siRNA for TAK1 or STO-609 on AMPK activation by hydroxyfasudil. AMPK activation by hydroxyfasudil was significantly decreased by siRNA for LKB1 and there was no additional effect by siRNA for TAK1 or STO-609 (**Fig. S12C,** **Fig. 6D**). Furthermore, siRNA for TAK1 or STO-609 per se had no effects on AMPK activation by hydroxyfasudil (**Fig. 6D**). These results indicate that LKB1 is substantially involved in the interaction between Rho-kinase and AMPK (**Fig. 7**).

## Discussion

The novel finding of the present study is that Rho-kinase inhibition ameliorates metabolic disorders through activation of the LKB1/AMPK pathway in mice (**Fig. 7**).

Because Rho-kinase enhances vascular smooth muscle proliferation, migration and contraction, its roles in the pathogenesis of atherosclerotic cardiovascular diseases attract much attention [5]–[7]. Furthermore, the role of Rho-kinase in the pathogenesis of metabolic disorders has also attracted much attention recently since Rho-kinase has been reported to be activated in metabolic syndrome in animals [5], [6] and humans [7]. Rho-kinase is negatively regulated by eNOS/NO pathway and visa versa [26]. Excessive calorie intake and low physical activity cause hypertension, obesity and insulin resistance, all of which cause endothelial dysfunction associated with down-regulation of eNOS/NO pathway and up-regulation of RhoA/Rho-kinase pathway, forming a vicious circle of metabolic disorders. However, since the detailed mechanism(s) of the relation between Rho-kinase and metabolic disorders has not been elucidated, we addressed this important issue in the present study. The present study provides a new insight into the mechanism by which Rho-kinase inhibition improves metabolic aberrations through activation of the LKB1/AMPKα2 pathway (**Fig. 7**). Although we showed that Rho-kinase inhibition improved metabolic aberrations through AMPK pathway, the only exception was for glucose metabolism as fasudil improved glucose metabolism even in AMPKα2^−/−^ mice in the present study. The acute effects of Rho-kinase inhibition on glucose metabolism are somewhat controversial. Rho-kinase phosphorylates insulin receptor substrate 1 (IRS1) and modulates insulin signal transduction either negatively or positively [27], [28]. However, the long-term inhibition of Rho-kinase in vivo exerts several beneficial effects on insulin resistance, such as suppression of inflammation, reduction in cytokines production and improvement of endothelial functions [2]–[4]. These findings could explain why long-term inhibition of Rho-kinase improved glucose metabolism in the present study. It was previously reported that systemic disruption of ROCK1, one of the isoforms of Rho-kinase, caused impaired insulin tolerance [29], while ROCK1 knockout mice specific for adipose tissue or hypothalamic arcuate neurons (POMC and AgRP) showed improved glucose metabolism compared with littermate control [30], [31]. Since in the present study, we inhibited Rho-kinase non-specifically by fasudil, the roles of each Rho-kinase isoform (ROCK1 and 2) in energy metabolism remain to be examined in further studies.

AMPK is a hetero-trimetric protein containing α, β and γ subunits and its activity is regulated by its phosphorylation at Thr172 and/or AMP/ATP ratio [32]. There are several up-stream kinases of AMPK, including LKB1 [23], CaMKKβ [24] and TAK1 [25]. LKB1 is also known as an energy sensor [33]. In the present study, we found that LKB1, but not CaMKKβ or TAK1, is substantially involved in the beneficial effects of Rho-kinase inhibition on AMPK activity (**Fig. 7**). When intracellular energy is starved, such as hypoxia [34], ischemia [35] and exercise [36], AMPK is activated, generating energy stock and stopping intracellular energy consumption [8]. Thus, AMPK works as an important energy sensor. Since AMPKα2, but not AMPKα1, is the major isoform in the skeletal muscle [37], we used AMPKα2^−/−^ mice and C2C12 myotubes in the present study. Indeed, we demonstrated that the fasudil treatment activates AMPKα2 in C2C12 myotubes, which could explain why fasudil was not effective in AMPKα2^−/−^ mice although some compensation by α1 isoform could be expected in the liver [15]. However, it was previously reported that Rho-kinase inhibition by fasudil increased rectal temperature in obese rats [6]. In the present study, we demonstrated that Rho-kinase inhibition increased energy expenditure through AMPK activation and increased mRNA expression of *Ucp1* and *Ppargc1a*, which are involved in thermogenesis in BAT. These results suggest that Rho-kinase inhibition also increases energy expenditure via AMPK activation in BAT. Thus, Rho-kinase inhibition could increase energy expenditure and body temperature.

### Interaction between Rho-kinase and AMP-knase

In the present study, we demonstrated that inhibition of Rho-kinase up-regulates the molecules that are linked to fatty acid oxidation (*CPT1a* and *Cpt1b*), mitochondrial energy production (*Cycs* and *Cox4i1*) and glucose transporter (*Slc2a4*) associated with improvement of metabolic phenotypes in vivo (**Fig. 7**). All these molecules are known as the downstream targets of AMPK (**Fig. 7**) [38], [39]. In addition, hydroxyfasudil also increased NAD^+^/NADH ratio, known as a downstream target of AMPK, is involved in Sirtuin-1 activity (**Fig. 7**) [10]. Since Sirtuin-1 exerts important anti-aging effects[40], these results suggest that Rho-kinase inhibition might also exert anti-aging effects. AMPK is activated in response to several pharmacological agents and some hormones, such as metformin [41], statins [42], resveratrol [43], 5-aminoimidazole-4-carboxamide-1-β-d- ribofuranoside [12], adiponectin [13] and leptin [44], exerting its beneficial effects on metabolic disorders. Metformin, an insulin sensitizer and activator of AMPK, reduces body weight and serum lipids, as does fasudil, in animals[45] and humans [46]. However, unlike fasudil, metformin does not affect blood pressure [47]. Although statins may inhibit Rho-kinase at supraclinical doses [47] and activate AMPK [41], they do not affect body weight or blood pressure [47] and might exacerbate glucose metabolism [48]. In contrast, fasudil ameliorates body weight [5], lipid profile [5], glucose metabolism [28] and blood pressure [49] via AMPK pathway. Thus, fasudil may be useful for the treatment of metabolic disorders.

### Study limitations

Several limitations should be mentioned for the present study. First, it remains to be elucidated which isoform of Rho-kinase (ROCK1 and ROCK2) mediates its inhibitory effects on AMPK. Second, since AMPK activation peaked at 6 hours in response to hydroxyfasudil, many steps could exist between Rho-kinase inhibition and AMPK activation. This point remains to be elucidated in future studies. Third, in the present study, we did not examine skeletal muscle fiber type distribution or measure fatty acid oxidation in isolated muscle. These issues remain to be addressed in future studies. Since AMPK is involved in skeletal muscle fiber type shift [50], this issue also remains to be examined in future studies. Fourth, the effect of fasudil on energy expenditure was not only skeletal muscle, because AMPK activity was increased in liver and the mRNA about energy expenditure was increased in BAT. This issue remains to be addressed in future studies using organ specific AMPK-KO mice. Finally, since fasudil and Y27632 are known to inhibit some other kinases such as PRK2 that is also one of Rho effector proteins at similar doses [51], all the beneficial effects of fasudil in the present study might not be mediated by Rho-kinase inhibition. This point also remains to be examined in future studies.

In conclusions, the present study demonstrates that Rho-kinase inhibition ameliorates metabolic disorders through activation of the LKB1/AMPK pathway in mice, suggesting that Rho-kinase could be a novel therapeutic target of metabolic disorders.

## Supporting Information

Figure S1 **Food Intake, Glucose Tolerance Test and Rho-kinase Activity of Wild-type Mice.** (**A**) Rho-kinase activity was measured by Western blotting in the white adipose tissue, liver and skeletal muscle. (**B**) There was no difference in food intake among the 3 groups. (**C**) Glucose tolerance test at 12-weeks of age showed that the responses were improved in the HFD-fas group compared with the HFD-cont group. Results are expressed as mean ± SEM. *P<0.05.
(TIF)

Figure S2 **Effects of Fasudil on Histological Changes and mRNA Expressions in Wild-type Mice.** (**A**) Representative photomicrographs of the WAT, BAT and liver (H&E staining) in the 3 wild-type mouse groups. Scale bar = 50 µm. (**B**) The size of BAT cells was significantly decreased in the HFD-fas group compared with the HFD-cont group. (**C**) mRNA expressions of *Ppargc1a* and *Ucp1* were significantly enhanced by the fasudil treatment. Results are expressed as mean ± SEM. *P<0.05.
(TIF)

Figure S3 **Metabolic Parameters in Wild-type Mice.** (**A**) Body weight at 8 weeks of age, (**B**) locomotor activity throughout the day and (**C**) respiration quotient were all comparable between the HFD-cont and the HFD-fas groups. Results are expressed as mean ± SEM.
(TIF)

Figure S4 **Improved Metabolic Phenotypes in Mice with Systemic Overexpression of Dominant-Negative Rho-kinase.** (**A**) The genotype of DN-ROCK Tg and littermate. DN-ROCK Tg had both DN-ROCK gene and CMV-Cre gene. (**B**) Rho-kinase activity of DN-ROCK Tg mice was approximately 30% less than that of littermate mice (female). (**C**) Food intake was comparable between the 2 groups compared with the HFD-littermate group (female). (**D**) Glucose tolerance test at 12-weeks of age showed that the responses were improved in the HFD-DN-ROCK Tg group compared with the HFD-littermate group. Results are expressed as mean ± SEM. *P<0.05 vs. HFD-DN-ROCK Tg group.
(TIF)

Figure S5 **Effects of Fasudil on Histological Changes and mRNA Expressions in Mice with Systemic Overexpression of Dominant-Negative Rho-kinase.** (**A**) Representative photomicrographs of the WAT, BAT and liver (H&E staining) in HFD-DN-ROCK Tg mice and HFD-littermate mice. Scale bar = 50 µm. (**B**) The size of BAT cells was significantly decreased in HFD-DN-ROCK Tg mice compared with HFD-littermate mice. (**C**) mRNA expressions of *Ppargc1a* and *Ucp1* were significantly enhanced in HFD-DN-ROCK Tg mice compared with HFD-littermate mice. (**D**) Mitochondrial DNA, as examined by real time RT-PCR as the ratio of mitochondrial DNA and nuclear DNA, was significantly increased in the HFD-DN-ROCK Tg mice compared with the littermate mice. Results are expressed as mean ± SEM. *P<0.05.
(TIF)

Figure S6 **Metabolic Parameters in AMPKα2^−/−^ Mice.** (**A**) AMPKα2 expression in the liver, skeletal muscle, white adipose tissue (WAT) and brown adipose tissue (BAT). AMPKα2^−/−^ mice lacked AMPKα2 protein. (**B**) Food intake was comparable between AMPKα2^−/−^ and littermate mice in both genders. (**C**) Glucose tolerance test at 12-weeks of age showed that the responses were improved in the HFD-AMPKα2^−/−^-fas group compared with the HFD-AMPKα2^−/−^-cont group in both genders. Results are expressed as mean ± SEM. *P<0.05.
(TIF)

Figure S7 **Food Intake and Glucose Tolerance Test in AMPKα2^−/−^ Mice.** (**A-C**) Body weight (**A**), locomotor activity (**B**), and respiration quotient (**C**) were all comparable between the HFD-AMPKα2^−/−^-cont and the HFD-AMPKα2^−/−^-fas groups (female). Results are expressed as mean ± SEM.
(TIF)

Figure S8 **Effects of Fasudil on Histological Changes and mRNA Expressions in AMPKα2^−/−^ Mice.** (**A**) Representative photomicrographs of the white adipose tissue (WAT), brown adipose tissue (BAT) and liver (H&E staining) in HFD-AMPKα2^−/−^-cont group and male HFD-AMPKα2^−/−^-fas group (male). Scale bar = 50 µm. (**B**) The size of BAT cells was comparable between the HFD-AMPKα2^−/−^-cont and male HFD-AMPKα2^−/−^-fas groups (male). (**C**) mRNA expressions of *Ppargc1a* and *Ucp1* in BAT was comparable between the 2 groups. (**D**) Mitochondrial DNA, measured by real time RT-PCR as the ratio of mitochondrial DNA and nuclear DNA, was comparable between the 2 groups. Results are expressed as mean ± SEM.
(TIF)

Figure S9 **Y27632 Activates AMPK in Vitro.** (**A**) In cultured C2C12 myotubes, Y-27632 (10 µmol/L) suppressed Rho-kinase activity and enhanced AMPK activity in a time-dependent manner. (**B**) Y-27632 inhibited Rho-kinase activity and enhanced AMPK activity in a concentration-dependent manner after 6 h of incubation. Results are expressed as mean ± SEM. *P<0.05.
(TIF)

Figure S10 **Fasudil Activate AMPKα2 But Not AMPKα1.** (**A**) Immunoprecipitation with AMPKα1 and AMPKα2 antibodies were performed in C2C12 myotubes. (**B**) Hydroxyfasudil treatment increased AMPK activity in whole cell lysate and samples immnoprecipitated with AMPKα2 but not in those with AMPKα1. Results are expressed as mean ± SEM. *P<0.05.
(TIF)

Figure S11 **Genetic Knockdown of Rho-kinase Activates AMPK in Vitro.** (**A**) siRNA for ROCK1 and ROCK2 inhibited protein expression of ROCK1 and ROCK2, respectively. (**B**) Combination of siROCK1 and siROCK2 significantly inhibited Rho-kinase activity and significantly enhanced AMPK activity. (**C**) The combination of siROCK1 and siROCK2 significantly up-regulated mRNA expression of downstream molecules of AMPK, Cpt1b and Cox4il, both of which were significantly inhibited by compound C. *P<0.05.
(TIF)

Figure S12 **Inhibitory Effects of siRNA on LKB1 and TAK1.** (**A**) siRNA for LKB1 inhibited protein expression of LKB1, whereas control siRNA was without effects. (**B**) siRNA for TAK1 inhibited protein expression of TAK1, whereas control siRNA was without effects. (**C**) The combination of siRNA for LKB1 and TAK1 inhibited protein expression of LKB1 and TAK1, respectively, whereas control siRNA was without effects.
(TIF)

Figure S13 **Rho-kinase Inhibits AMPK Activity Through LKB1 Pathway.** (**A**) Hydroxyfasudil suppressed Rho-kinase activity and siRNA for LKB1 inhibited hydroxyfasudil-induced AMPK phosphorylation. (**B**) Hydroxyfasudil suppressed Rho-kinase activity and increased phosphorylation of AMPK with or without a CaMKKβ inhibitor, STO-609. Results are expressed as mean ± SEM. *P<0.05.
(TIF)

Figure S14 **Rho-kinase Inhibits AMPK Activity via LKB1 Pathway.** (**A**) Hydroxyfasudil suppressed Rho-kinase activity and increased AMPK activity with or without siRNA for TAK1. (**B**) Hydroxyfasudil suppressed Rho-kinase activity and increased AMPK activity with or without a TAK1 inihibitor, oxozeaenol. Results are expressed as mean ± SEM. *P<0.05.
(TIF)

Table S1 **Sequence of siRNAs (Qiagen).** The sequence of siRNAs for ROCK1, ROCK2, LKB1 and TAK1.
(PDF)
