# Supplementary figures and images for: Rho-Kinase Inhibition Ameliorates Metabolic Disorders through Activation of AMPK Pathway in Mice
Source: PLoS One. 2014 Nov 3;9(11):e110446. doi: 10.1371/journal.pone.0110446 (PMC4217731; doi:10.1371/journal.pone.0110446)

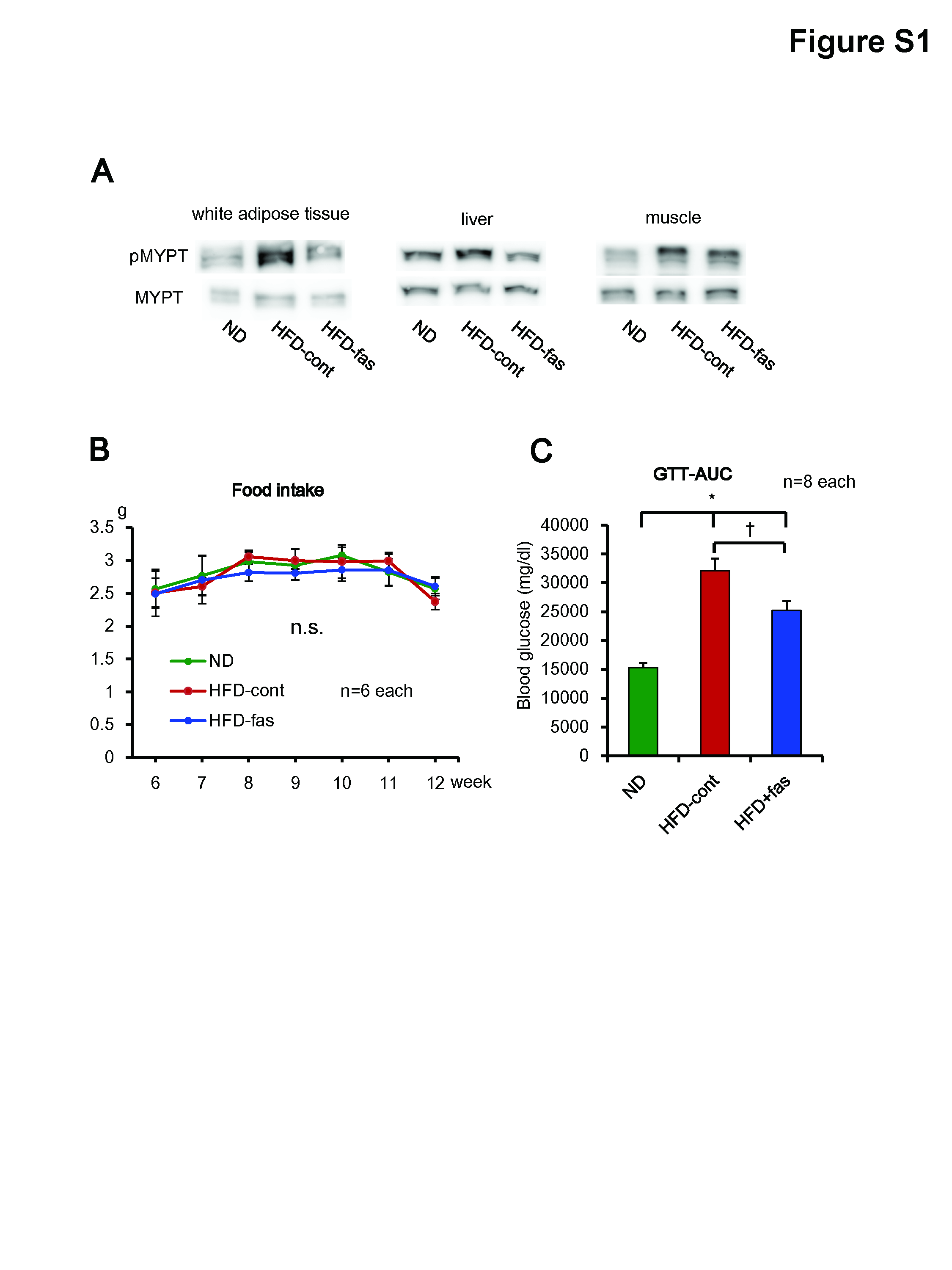

Supplement: Figure S1 — Food Intake, Glucose Tolerance Test and Rho-kinase Activity of Wild-type Mice. (A) Rho-kinase activity was measured by Western blotting in the white adipose tissue, liver and skeletal muscle. (B) There was no difference in food intake among the 3 groups. (C) Glucose tolerance test at 12-weeks of age showed that the responses were improved in the HFD-fas group compared with the HFD-cont group. Results are expressed as mean ± SEM. *P<0.05. (TIF) [file pone.0110446.s001.tif]

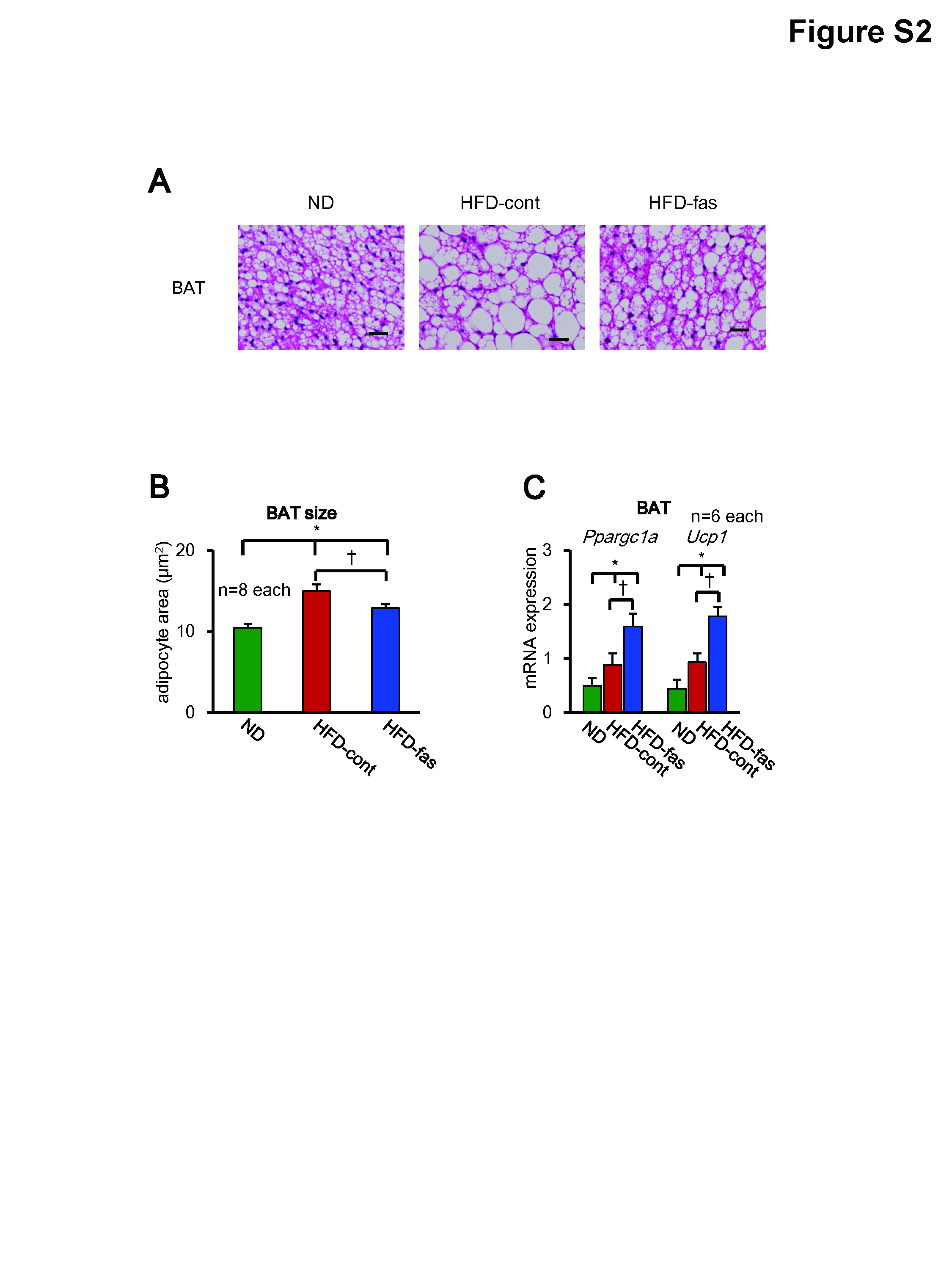

Supplement: Figure S2 — Effects of Fasudil on Histological Changes and mRNA Expressions in Wild-type Mice. (A) Representative photomicrographs of the WAT, BAT and liver (H&E staining) in the 3 wild-type mouse groups. Scale bar = 50 µm. (B) The size of BAT cells was significantly decreased in the HFD-fas group compared with the HFD-cont group. (C) mRNA expressions of Ppargc1a and Ucp1 were significantly enhanced by the fasudil treatment. Results are expressed as mean ± SEM. *P<0.05. (TIF) [file pone.0110446.s002.tif]

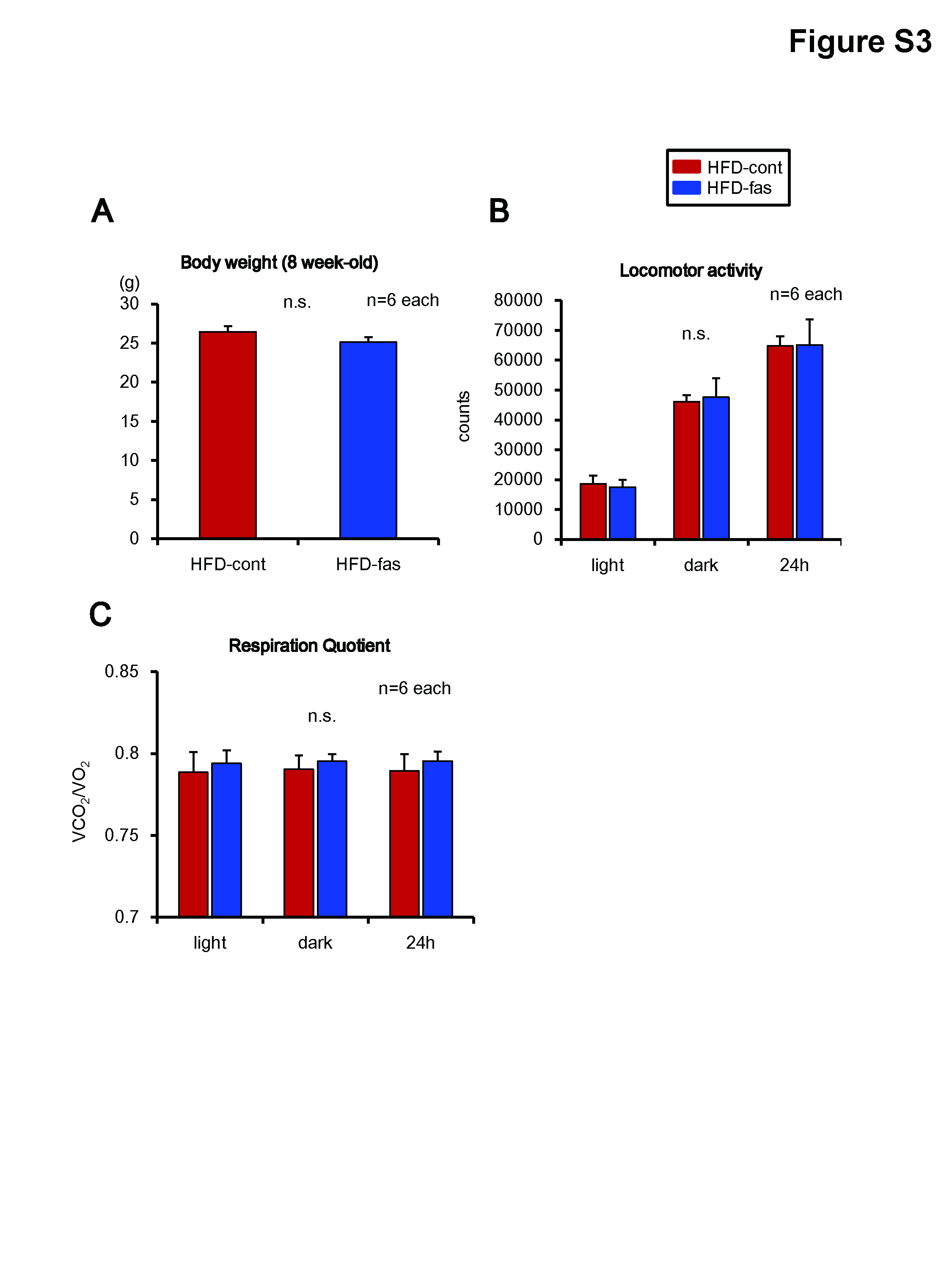

Supplement: Figure S3 — Metabolic Parameters in Wild-type Mice. (A) Body weight at 8 weeks of age, (B) locomotor activity throughout the day and (C) respiration quotient were all comparable between the HFD-cont and the HFD-fas groups. Results are expressed as mean ± SEM. (TIF) [file pone.0110446.s003.tif]

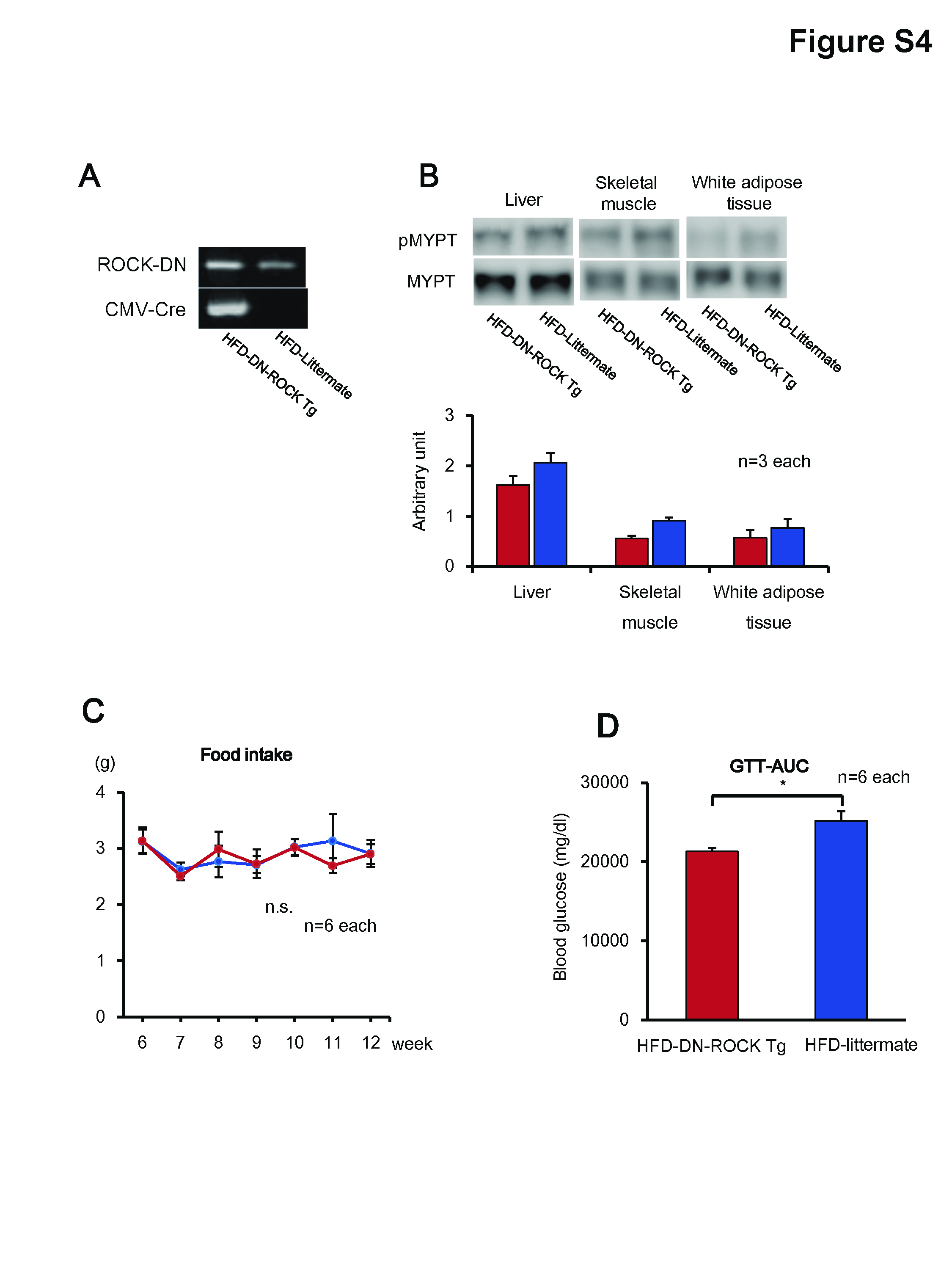

Supplement: Figure S4 — Improved Metabolic Phenotypes in Mice with Systemic Overexpression of Dominant-Negative Rho-kinase. (A) The genotype of DN-ROCK Tg and littermate. DN-ROCK Tg had both DN-ROCK gene and CMV-Cre gene. (B) Rho-kinase activity of DN-ROCK Tg mice was approximately 30% less than that of littermate mice (female). (C) Food intake was comparable between the 2 groups compared with the HFD-littermate group (female). (D) Glucose tolerance test at 12-weeks of age showed that the responses were improved in the HFD-DN-ROCK Tg group compared with the HFD-littermate group. Results are expressed as mean ± SEM. *P<0.05 vs. HFD-DN-ROCK Tg group. (TIF) [file pone.0110446.s004.tif]

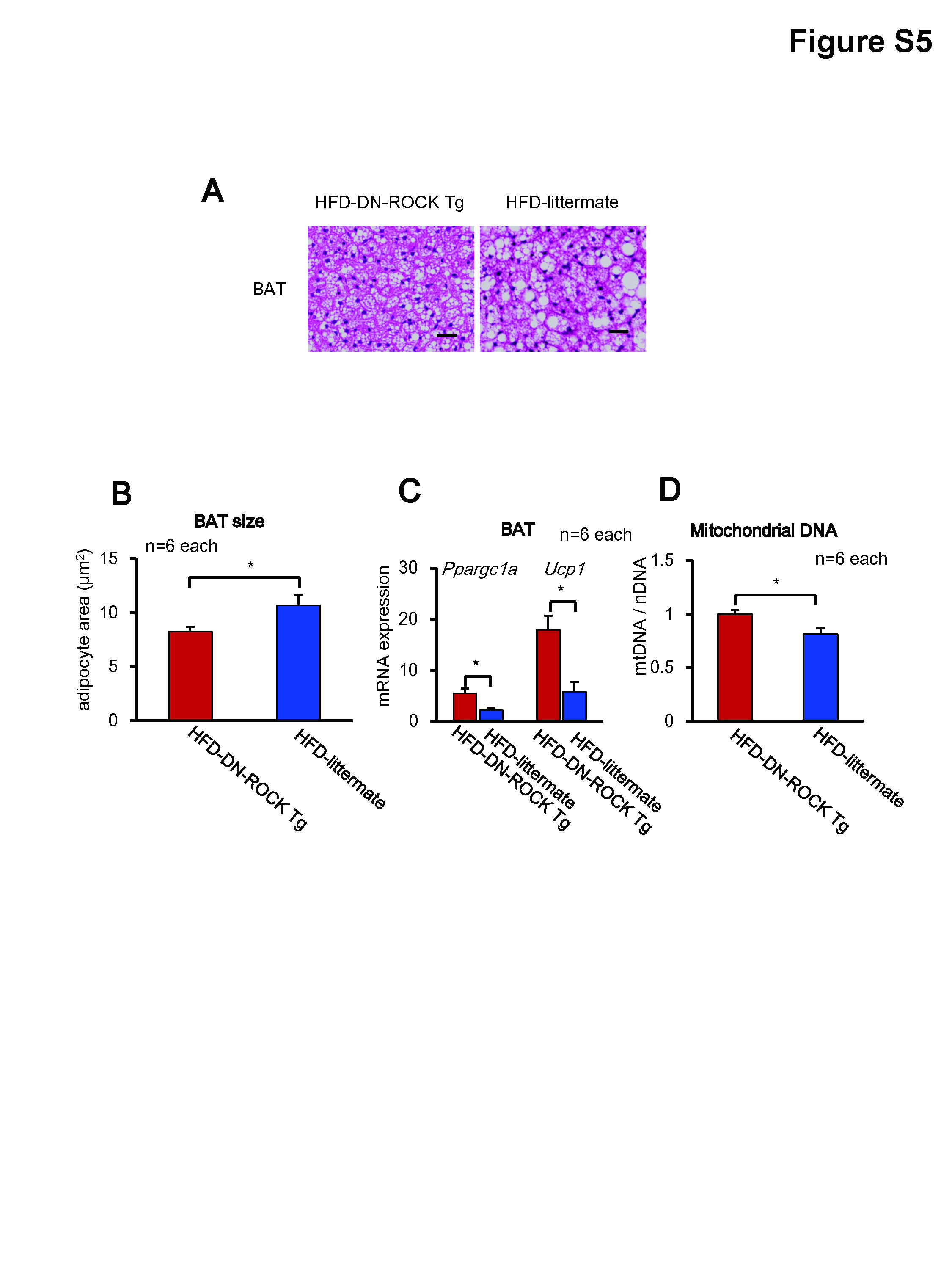

Supplement: Figure S5 — Effects of Fasudil on Histological Changes and mRNA Expressions in Mice with Systemic Overexpression of Dominant-Negative Rho-kinase. (A) Representative photomicrographs of the WAT, BAT and liver (H&E staining) in HFD-DN-ROCK Tg mice and HFD-littermate mice. Scale bar = 50 µm. (B) The size of BAT cells was significantly decreased in HFD-DN-ROCK Tg mice compared with HFD-littermate mice. (C) mRNA expressions of Ppargc1a and Ucp1 were significantly enhanced in HFD-DN-ROCK Tg mice compared with HFD-littermate mice. (D) Mitochondrial DNA, as examined by real time RT-PCR as the ratio of mitochondrial DNA and nuclear DNA, was significantly increased in the HFD-DN-ROCK Tg mice compared with the littermate mice. Results are expressed as mean ± SEM. *P<0.05. (TIF) [file pone.0110446.s005.tif]

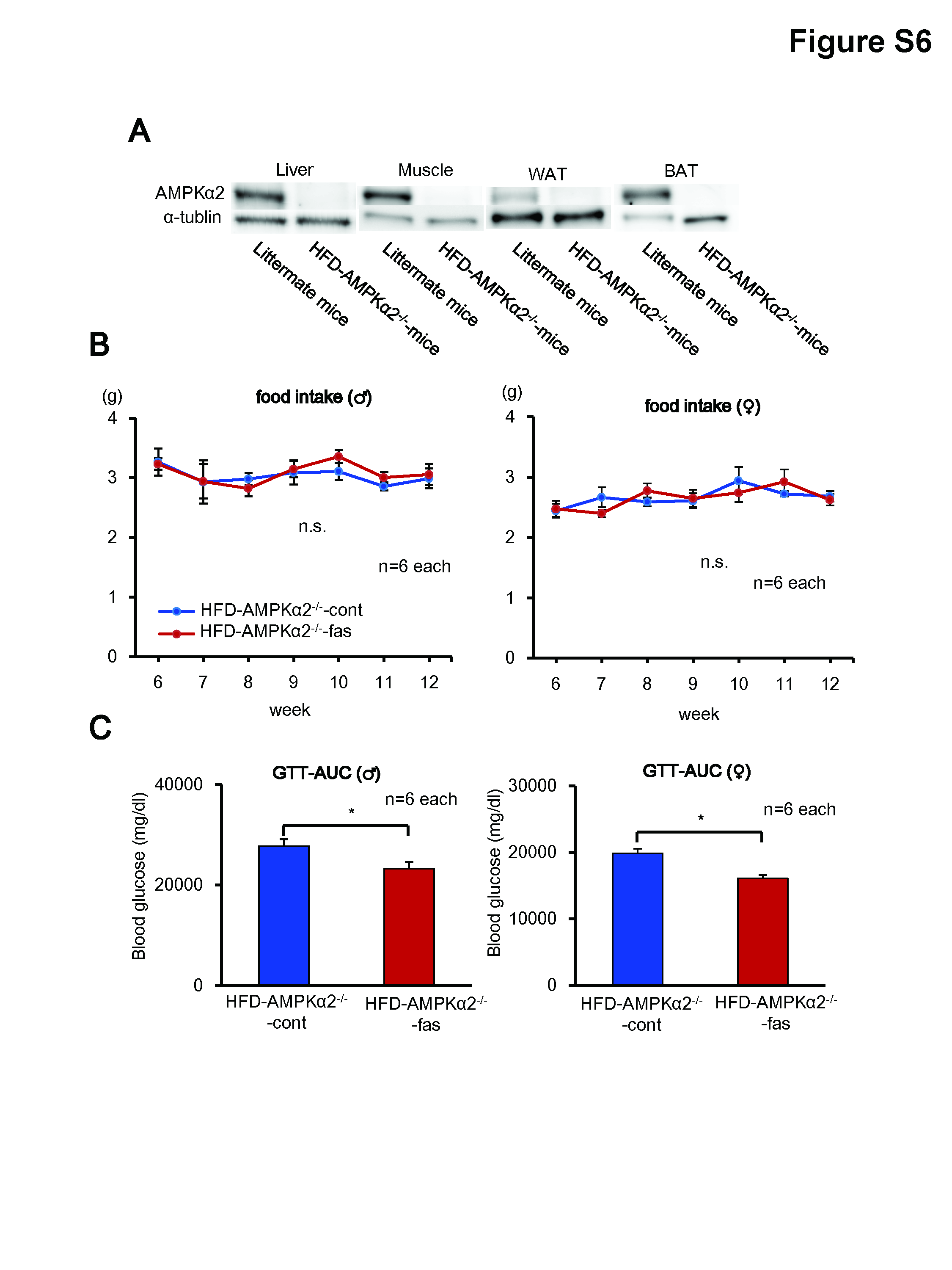

Supplement: Figure S6 — Metabolic Parameters in AMPKα2−/− Mice. (A) AMPKα2 expression in the liver, skeletal muscle, white adipose tissue (WAT) and brown adipose tissue (BAT). AMPKα2−/− mice lacked AMPKα2 protein. (B) Food intake was comparable between AMPKα2−/− and littermate mice in both genders. (C) Glucose tolerance test at 12-weeks of age showed that the responses were improved in the HFD-AMPKα2−/−-fas group compared with the HFD-AMPKα2−/−-cont group in both genders. Results are expressed as mean ± SEM. *P<0.05. (TIF) [file pone.0110446.s006.tif]

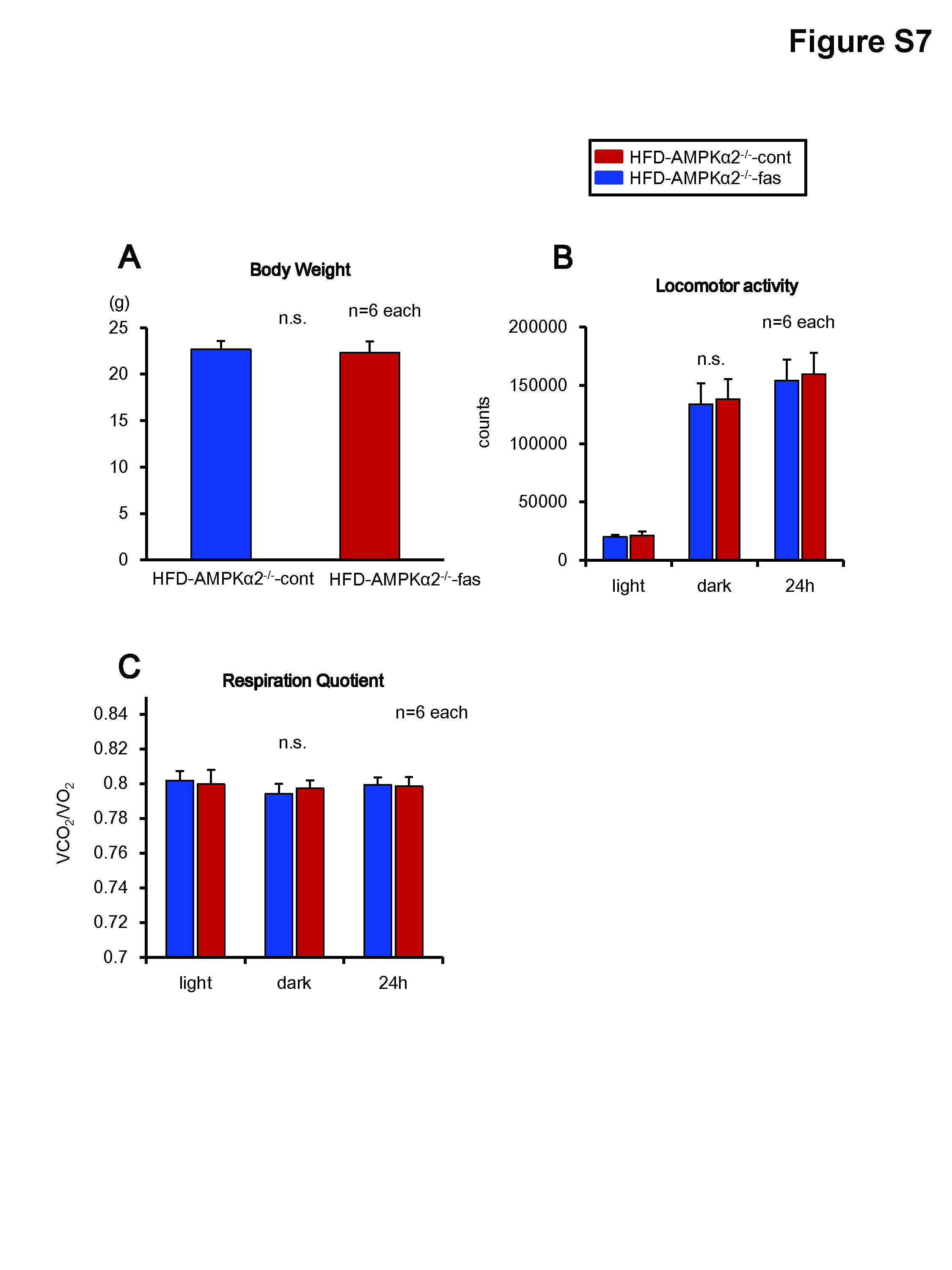

Supplement: Figure S7 — Food Intake and Glucose Tolerance Test in AMPKα2−/− Mice. (A-C) Body weight (A), locomotor activity (B), and respiration quotient (C) were all comparable between the HFD-AMPKα2−/−-cont and the HFD-AMPKα2−/−-fas groups (female). Results are expressed as mean ± SEM. (TIF) [file pone.0110446.s007.tif]

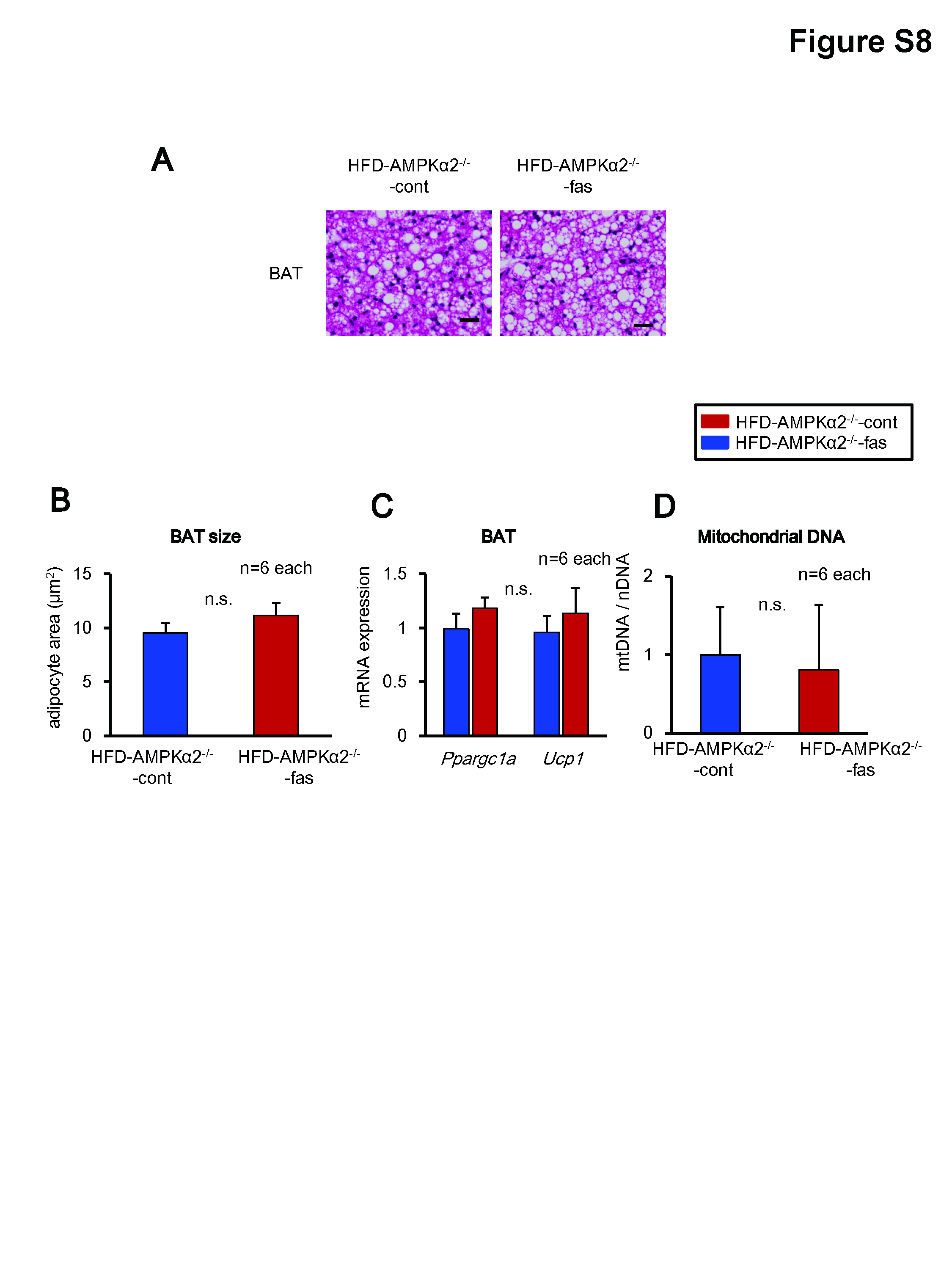

Supplement: Figure S8 — Effects of Fasudil on Histological Changes and mRNA Expressions in AMPKα2−/− Mice. (A) Representative photomicrographs of the white adipose tissue (WAT), brown adipose tissue (BAT) and liver (H&E staining) in HFD-AMPKα2−/−-cont group and male HFD-AMPKα2−/−-fas group (male). Scale bar = 50 µm. (B) The size of BAT cells was comparable between the HFD-AMPKα2−/−-cont and male HFD-AMPKα2−/−-fas groups (male). (C) mRNA expressions of Ppargc1a and Ucp1 in BAT was comparable between the 2 groups. (D) Mitochondrial DNA, measured by real time RT-PCR as the ratio of mitochondrial DNA and nuclear DNA, was comparable between the 2 groups. Results are expressed as mean ± SEM. (TIF) [file pone.0110446.s008.tif]

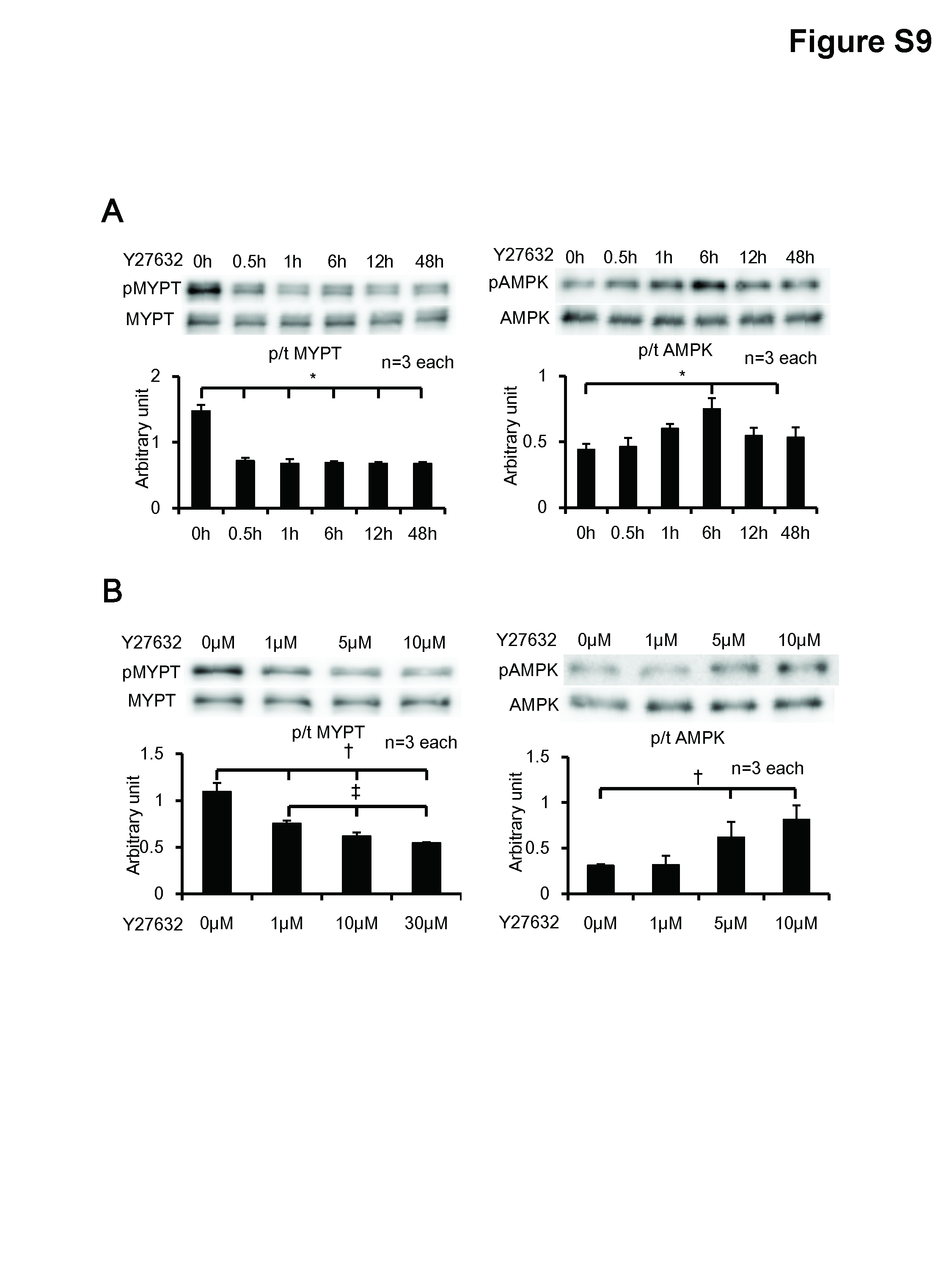

Supplement: Figure S9 — Y27632 Activates AMPK in Vitro. (A) In cultured C2C12 myotubes, Y-27632 (10 µmol/L) suppressed Rho-kinase activity and enhanced AMPK activity in a time-dependent manner. (B) Y-27632 inhibited Rho-kinase activity and enhanced AMPK activity in a concentration-dependent manner after 6 h of incubation. Results are expressed as mean ± SEM. *P<0.05. (TIF) [file pone.0110446.s009.tif]

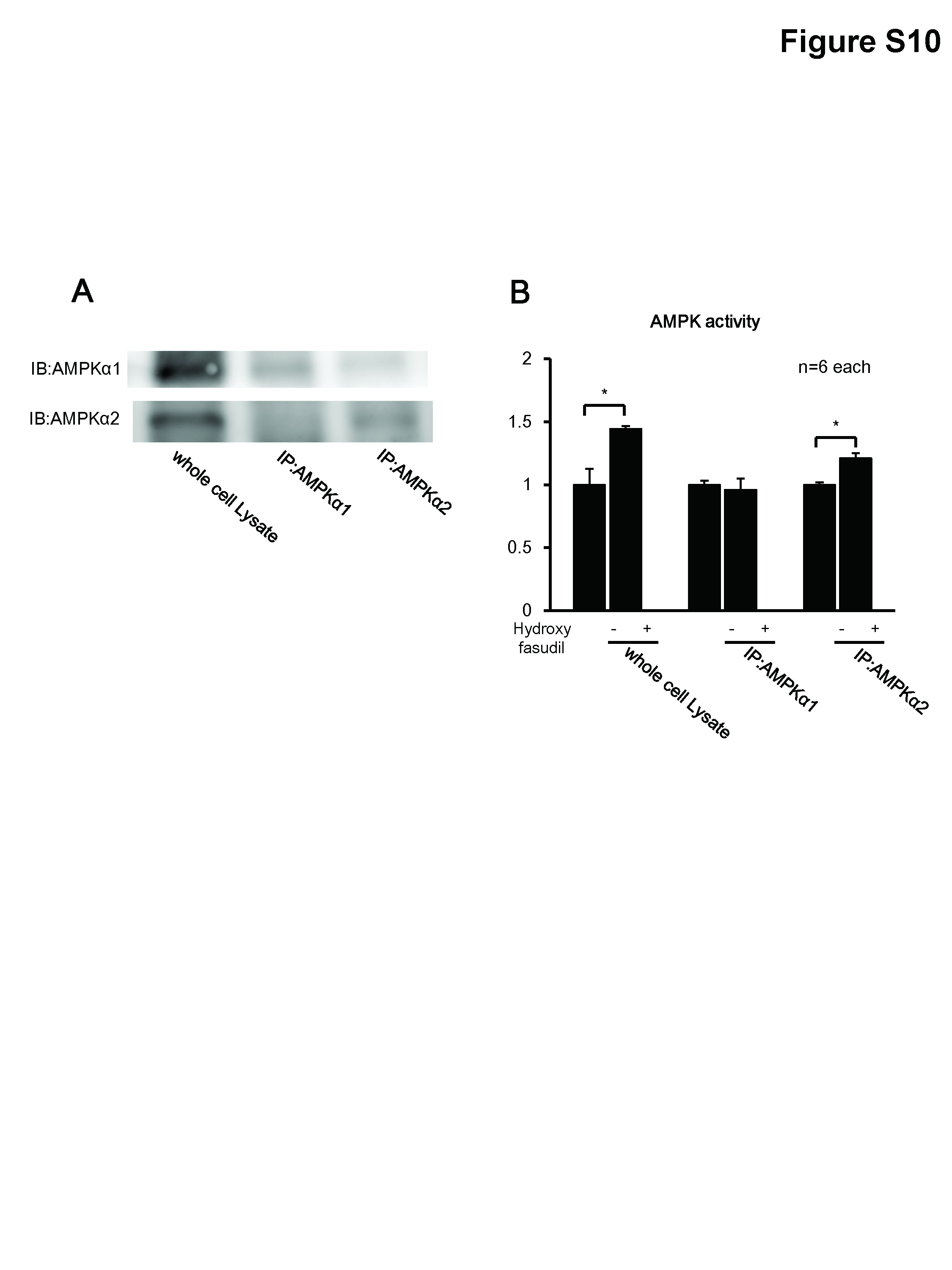

Supplement: Figure S10 — Fasudil Activate AMPKα2 But Not AMPKα1. (A) Immunoprecipitation with AMPKα1 and AMPKα2 antibodies were performed in C2C12 myotubes. (B) Hydroxyfasudil treatment increased AMPK activity in whole cell lysate and samples immnoprecipitated with AMPKα2 but not in those with AMPKα1. Results are expressed as mean ± SEM. *P<0.05. (TIF) [file pone.0110446.s010.tif]

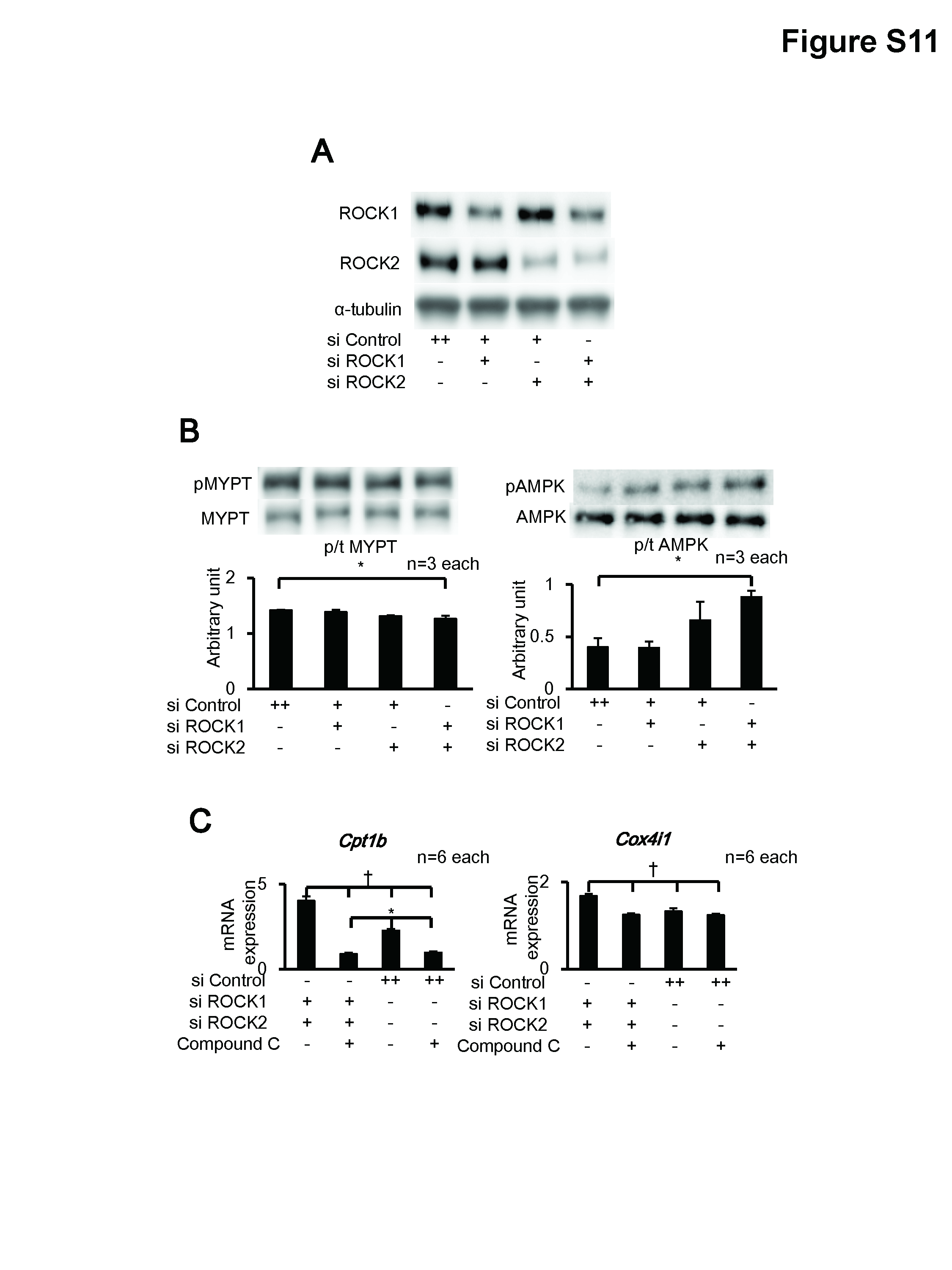

Supplement: Figure S11 — Genetic Knockdown of Rho-kinase Activates AMPK in Vitro. (A) siRNA for ROCK1 and ROCK2 inhibited protein expression of ROCK1 and ROCK2, respectively. (B) Combination of siROCK1 and siROCK2 significantly inhibited Rho-kinase activity and significantly enhanced AMPK activity. (C) The combination of siROCK1 and siROCK2 significantly up-regulated mRNA expression of downstream molecules of AMPK, Cpt1b and Cox4il, both of which were significantly inhibited by compound C. *P<0.05. (TIF) [file pone.0110446.s011.tif]

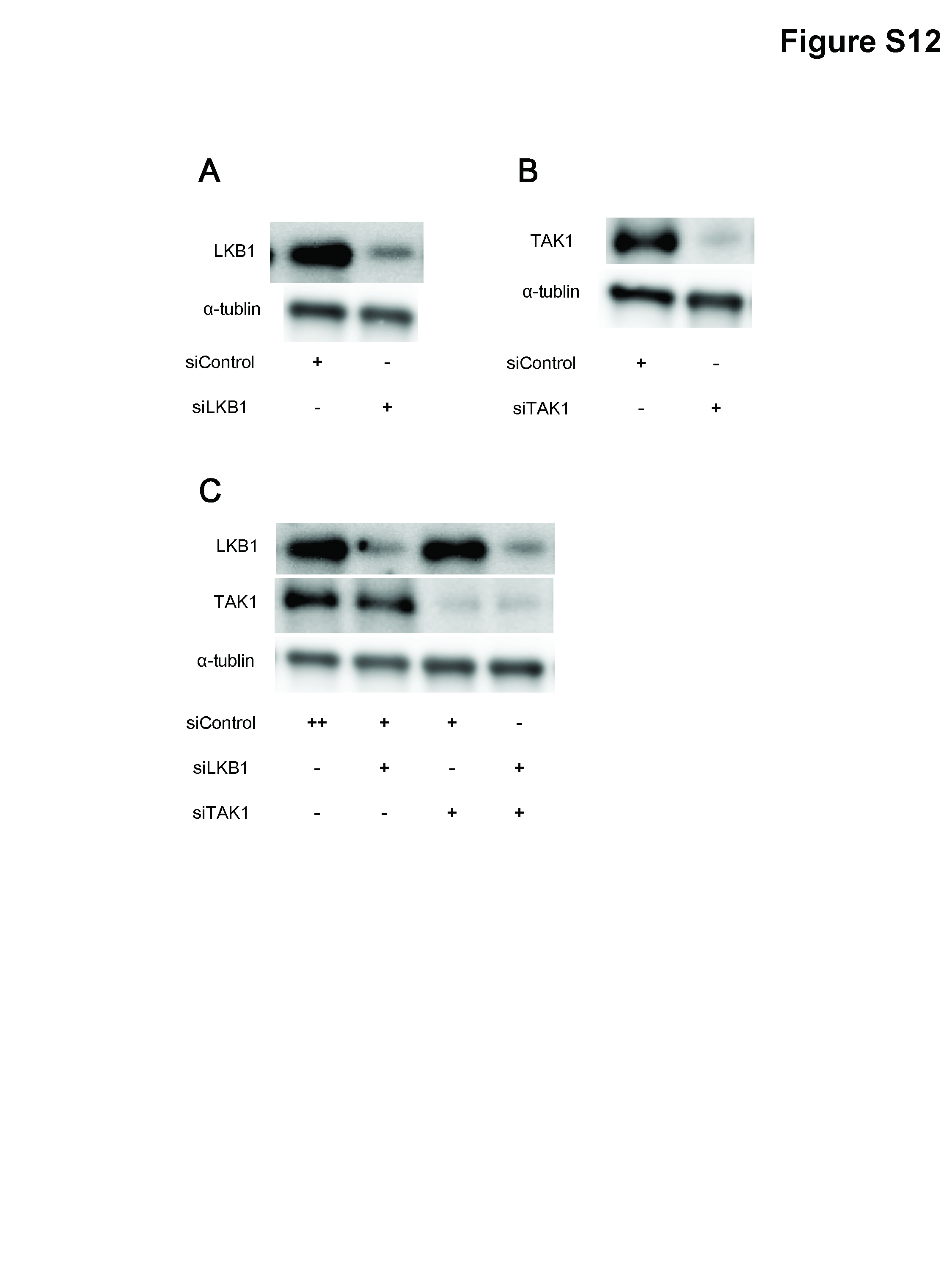

Supplement: Figure S12 — Inhibitory Effects of siRNA on LKB1 and TAK1. (A) siRNA for LKB1 inhibited protein expression of LKB1, whereas control siRNA was without effects. (B) siRNA for TAK1 inhibited protein expression of TAK1, whereas control siRNA was without effects. (C) The combination of siRNA for LKB1 and TAK1 inhibited protein expression of LKB1 and TAK1, respectively, whereas control siRNA was without effects. (TIF) [file pone.0110446.s012.tif]

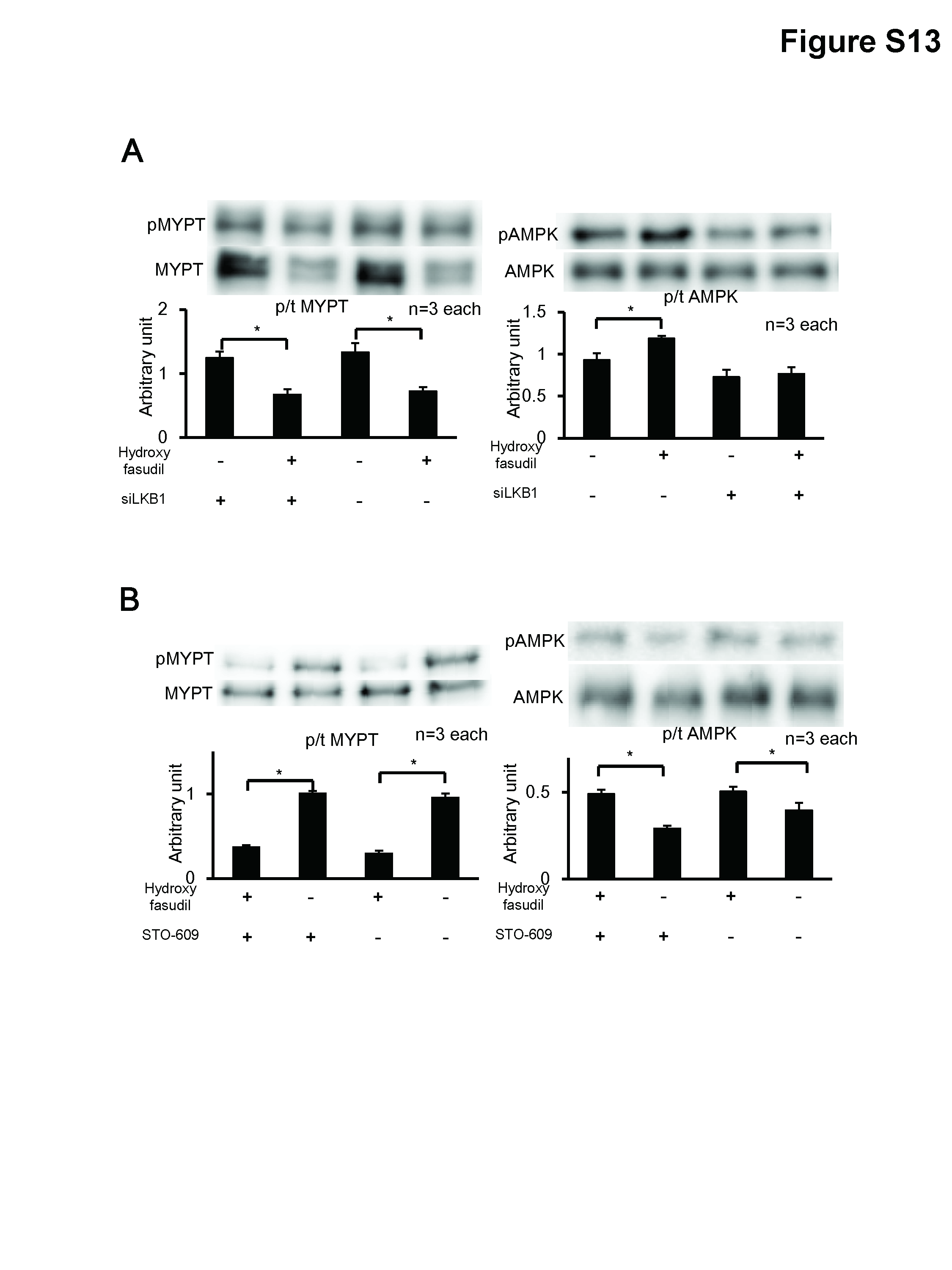

Supplement: Figure S13 — Rho-kinase Inhibits AMPK Activity Through LKB1 Pathway. (A) Hydroxyfasudil suppressed Rho-kinase activity and siRNA for LKB1 inhibited hydroxyfasudil-induced AMPK phosphorylation. (B) Hydroxyfasudil suppressed Rho-kinase activity and increased phosphorylation of AMPK with or without a CaMKKβ inhibitor, STO-609. Results are expressed as mean ± SEM. *P<0.05. (TIF) [file pone.0110446.s013.tif]

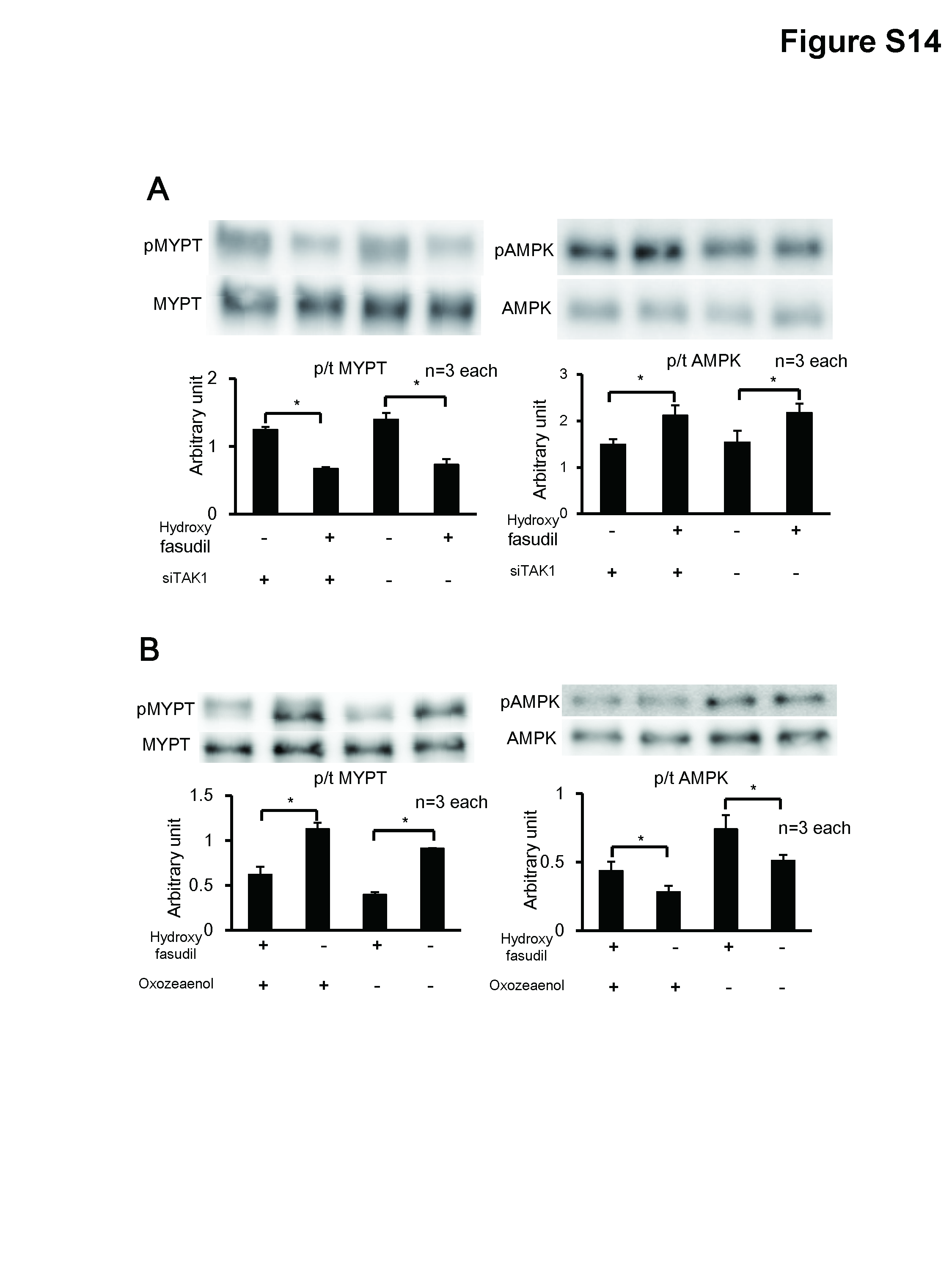

Supplement: Figure S14 — Rho-kinase Inhibits AMPK Activity via LKB1 Pathway. (A) Hydroxyfasudil suppressed Rho-kinase activity and increased AMPK activity with or without siRNA for TAK1. (B) Hydroxyfasudil suppressed Rho-kinase activity and increased AMPK activity with or without a TAK1 inihibitor, oxozeaenol. Results are expressed as mean ± SEM. *P<0.05. (TIF) [file pone.0110446.s014.tif]
