# Supplementary material for: Rho-Kinase Inhibition Ameliorates Metabolic Disorders through Activation of AMPK Pathway in Mice
Source: PLoS One. 2014 Nov 3;9(11):e110446. doi: 10.1371/journal.pone.0110446 (PMC4217731; doi:10.1371/journal.pone.0110446)
Supplement: Table S1 — Sequence of siRNAs (Qiagen). The sequence of siRNAs for ROCK1, ROCK2, LKB1 and TAK1. (PDF) [file pone.0110446.s015.pdf]

Online-Only Data Supplement

Supplemental Tables

Table S1. Sequence of siRNAs (Qiagen)

Gene symbol	Sequence (sense)
Rock1	CAAGAAGTAAATGAACATAAA
Rock2	GGAUAAACAUGGACAUCUATT
LKB1	CACCACCAATGGCACACTCAA
TAK1	TCGGTCTGTTATACCAAATAA
